# Cell differentiation can underpin the reproducibility of morphogenesis

**DOI:** 10.1371/journal.pcbi.1014361

**Published:** 2026-06-04

**Authors:** Dominic K. Devlin, Austen R. D. Ganley, Nobuto Takeuchi

**Affiliations:** 1 School of Biological Sciences, University of Auckland, Auckland, New Zealand; 2 Graduate School of Arts and Sciences, The University of Tokyo, Tokyo, Japan; 3 Universal Biology Institute, The University of Tokyo, Tokyo, Japan; 4 Department of Biology, Faculty of Sciences, Kyushu University, Fukuoka, Japan; Chinese Academy of Sciences, CHINA

## Abstract

Morphogenesis of complex body shapes is reproducible despite the noise inherent in the underlying morphogenetic processes. However, how these morphogenetic processes work together to achieve this reproducibility remains unclear. Here, we ask how this reproducibility is achieved by evolving complex morphologies in a multi-scale, computational model. Each morphology consists of a population of cells on a two-dimensional grid using the Cellular Potts Model framework. Each cell contains a genome that encodes a gene regulatory network, morphogens for cell-cell signalling, and proteins that determine cell behaviours. By repeatedly simulating our model with different initial conditions under selection for shape complexity, we obtained a “zoo” of evolved morphologies. We find that these evolved, complex morphologies are reproducible in a sizeable fraction of simulations, despite no direct selection for reproducibility. We show that high reproducibility is caused by spatially segregating moving cells that “shape” morphologies from stationary cells that “maintain” morphologies during morphogenesis. Strikingly, most highly reproducible morphologies also evolved cell differentiation, where proliferative, moving progenitor cells irreversibly differentiate into non-dividing, stationary differentiated cells at tissue boundaries. These results suggest that cell differentiation observed in natural development plays a fundamental role in morphogenesis in addition to the production of specialised cell types. This previously unrecognised role of cell differentiation has major implications for our understanding of how morphologies are generated and regenerated.

## Author summary

An astounding property of multicellular animals is the reliable formation of morphological structures, such as organs and limbs. How does this reliable formation, termed reproducibility, arise despite the inherent noise of biological systems? To understand the origins of reproducibility, we developed a computational model of multicellular morphologies. We applied an evolutionary algorithm to our model to search for different kinds of complex morphologies. We found that a significant fraction of these evolved morphologies were highly reproducible, even though we did not select for reproducibility. We showed that reproducibility emerges through a division of labour between moving, shape-making cells and static, shape-maintaining cells. Strikingly, the shape-making cells almost always irreversibly differentiate into shape-maintaining cells—a process that mirrors natural multicellular development. Our findings suggest that irreversible cell differentiation plays an important role in making reproducible morphologies. Our results may be relevant to improving the reproducibility of *in vitro* tissues such as organoids, a current challenge in tissue bioengineering.

## Introduction

Morphogenesis is the multifaceted process that transforms relatively homogeneous starting materials—such as a zygote or group of progenitor cells—into the complex morphological structures, such as organs, tissues and appendages, that constitute a mature organism [1,2]. This transformation occurs through a combination of chemical-level pattern formation and cellular-level shape formation [3,4]. At the chemical level, reacting and diffusing chemicals produce spatial patterns, such as stripes and segments [5,6]. At the cellular-level, processes such as cell motion, division, contraction and differential adhesion interact with chemical-level pattern formation to produce morphological shapes, such as tails, tubes, branches and limbs [1,2,7–9]. For extant animals, the morphological structures produced by these processes are not only complex, but also reproduced across generations. Understanding how complex morphogenesis is made reproducible has intrigued the minds of thinkers since Pythagoras and Aristotle [10], and is a focal point of developmental biology [4,11–13].

The reproducibility of morphogenesis requires both cell-level and chemical-level processes to be robust to noise [14,15]. Much attention has been devoted to understanding the robustness of chemical-level pattern formation to molecular sources of noise, such as fluctuations in chemical concentrations [16], resulting in the characterisation of chemical-level processes that enhance pattern reproducibility, such as genetic feedback loops and signal transduction pathways [17–24]. In contrast, much less is known about the cellular processes underlying the robustness of morphogenesis to cell-level sources of noise, such as stochasticity in the motion and geometry of cells [25]. To address this issue, previous studies have taken a targeted approach in which they selected a set of cell-level processes, such as cell-cell signalling and adhesion between cell types, and examined each process to determine whether it increases morphogenetic reproducibility [26,27].

Here, we instead asked whether morphogenetic reproducibility is an emergent by-product of morphogenesis that evolves even if reproducibility is not explicitly selected for, and, if so, how this reproducibility is realised—a non-prescriptive approach developed by Hogeweg, Ten Tusscher & Vroomans [28–33]. To determine whether reproducibility is a by-product of morphogenesis, we computationally generated an ensemble of “morphogeneses” by repeatedly evolving morphologies selected for geometrically complex shapes. We found that a sizeable fraction of morphologies was much more reproducible than others, even though reproducibility was not explicitly selected. Strikingly, these morphologies shared one cell-level feature responsible for morphogenetic reproducibility: a “morphogenetic division of labour”, where moving and dividing progenitor cells “shape” morphologies, while non-moving and non-dividing differentiated cells spatially “anchor” this shaping process, thereby enhancing morphogenetic reproducibility.

## Results

### Multi-scale model

**Fig 1:**
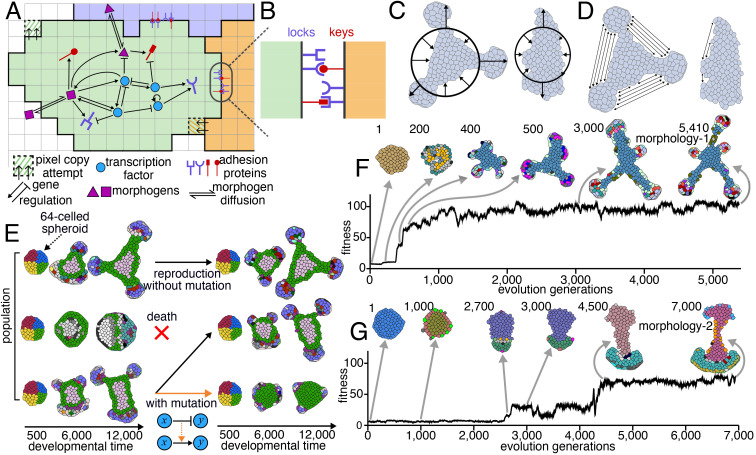
Multi-scale model of morphogenesis evolution. **(A)** Three neighbouring cells on a CPM grid. A cell consists of one or more pixels. Each cell is coloured by its “cell state” defined by the concentrations of all its proteins converted to Boolean values (the medium is represented by white pixels). Pixels with alternating stripes indicate pixel copies at cell boundaries. Each cell contains a genome that encodes transcription factors (TFs; circles, squares, triangles), adhesion proteins (sticks with a lock or key) and membrane tension proteins (not shown). Arrows indicate regulation of gene expression by TFs (arrow head for activation, blunt head for inhibition). Double harpoon arrows indicate diffusion of morphogens (membrane-permeable TFs). **(B)** Adhesion proteins facilitate the binding of cells to each other via a lock and key mechanism, or to the surrounding medium (not shown). **(C, D)** Illustration of shape complexity measurements for two morphologies. (C) depicts the measurement of how much the morphology deviates from a perfect circle (black), with the centre of the circle being the morphology’s centre of mass. Black arrows at equidistant intervals around the circle mark directions where the morphology’s shape deviates from the circle, with longer arrows contributing to a higher score. (D) depicts the measurement of inward folding using double sided arrows, with more arrows contributing to a higher score. The cells are all coloured grey to emphasise that only the shape, not cell states, determine the complexity score. **(E)** A population consists of 60 morphologies (three depicted). Morphologies undergo a developmental phase on separate CPM grids for 12,000 DTS, and then a reproduction phase, where morphologies with complex shapes are selected. Reproduction can occur without mutation (black arrows) or with mutation (orange arrow), with mutation determined probabilistically. Mutations change the topology of the GRN (dashed orange arrow), with the example showing a change from inhibition of gene *y* by gene *x* to activation of gene *y* by gene ***x*. (F, G)** Five-generation moving average of population fitness (shape complexity) over two separate evolutionary simulations. The morphologies above the plots are a trace of ancestors, with each morphology depicted at the end of its development (12,000 DTS) from six different generations over the course of evolution. The generation number of each morphology is shown to its left. “Evolved morphologies” of each simulation are shown on the far right.

To investigate whether reproducibility is a by-product of complex morphogenesis, we constructed a deliberately simplified model of morphogenesis. To capture the noisy dynamics of real morphogenesis, we employed the Cellular Potts Model (CPM), which uses a Metropolis algorithm to simulate stochastic cell motion and cell shape dynamics [34,35]. The CPM models the development of a group of cells on a two-dimensional square grid (250 × 250 pixels). The population of cells on the grid represents a developing tissue, which we term a “morphology”. Each cell consists of a collection of neighbouring pixels on the grid (Fig 1A). Pixels not occupied by cells represent the medium (Fig 1A), which is akin to an extracellular matrix or fluid [36]. Cell motion occurs through stochastic extensions and retractions of cell boundaries driven by pixel copy processes at these boundaries (Fig 1A; see Methods). Pixels on the grid are chosen in a random order with replacement for copy attempts. The unit of time is the number of pixel copy attempts equal to the total number of pixels on the grid, hereafter referred to as a developmental time step (DTS). The probability that a pixel copy occurs is determined by the minimisation of free energy arising from cell-cell adhesion, cell-medium adhesion, cell shape and cell size, as described later.

To simulate the development of a morphology, the CPM is run for 12,000 DTS, which is approximately the minimum time it takes for a fast-growing morphology to reach the edge of the grid. Each morphology starts as a circular shape consisting of 64 cells of approximately 75 pixels each (Fig 1E). After initialisation, cells grow if they are mechanically stretched and shrink if they are mechanically squeezed (see Materials and methods). Cell stretching and squeezing are induced by adhesion to neighbouring cells and the extracellular medium, although they can also occur stochastically. When a cell reaches a size of 100 pixels, it divides into two daughter cells of approximately equal size. The plane of cell division aligns with the minor axis of the mother cell, thereby minimising the elongation of the resulting daughter cells. Linking cell division to stretching is a simplified mechanism of growth reflecting the mechano-sensitive cell division observed in several developmental contexts [37–39].

To model basic cell mechanics, we equip cells with genes encoding three kinds of proteins that are nearly universally present in animal cells during development: adhesion proteins, membrane tension proteins and transcription factors. Adhesion proteins modulate the adhesiveness of cells to neighbouring cells and the extracellular medium (Fig 1A and 1B). These proteins are modelled as either locks or keys. The adhesion energy between two neighbouring cells is proportional to the number of complementary lock-key pairs they express. Similarly, the adhesion energy of a cell to the medium is proportional to the number of medium-adhesion proteins expressed by that cell (Materials and methods). Differential adhesion energies drive the rearrangement of cells to maximise contact with other cells or the medium to which they strongly adhere (small energy) and minimise contact with those to which they weakly adhere (large energy), thereby inducing directed cell motion. Adhesion proteins also influence cell fluidity, i.e., the rate of cell rearrangements. When the free energy arising from cell-cell adhesion is low, cells behave more fluid-like (e.g., mesenchymal cells). When this energy is high, cells behave more solid-like (e.g., epithelial cells) [40]. Differential adhesion is incorporated into our model, as it is known to drive several processes essential for real morphogenesis, such as cell sorting, formation of tissue boundaries and cell migration [2,41].

Membrane tension proteins make the cell shape less deformable by energetically constraining the length of its dynamically determined longest axis, irrespective of the cell’s orientation. By making the cell shape less deformable, membrane tension proteins suppress cell motion. The constraint on the length of the longest axis increases with the number of expressed tension proteins (Materials and methods). These proteins model an increase in cell membrane tension, which occurs in real cells by the accumulation of actin filament stress [42]. To maintain the simplicity and broad applicability of the model, we do not implement polarised cell tension or contractility, properties required for processes like invagination.

To model gene regulation, we couple each CPM cell to gene expression dynamics, as previously done [29,30,43–46]. Each cell is equipped with transcription factors (TFs), that form a gene regulatory network (GRN) with its adhesion and membrane tension proteins. A GRN is a graph consisting of nodes representing proteins and edges representing TF-mediated activation or inhibition of gene expression (Fig 1A). Concentrations of proteins within a cell are determined by numerically integrating a set of ordinary differential equations given by the GRN (Materials and methods). To model cell-cell signalling, a minority fraction of TFs (hereafter called morphogens) diffuse between cells and into the medium (Fig 1A; Materials and methods). These morphogens model those encountered in real development, such as Wnts and BMPs [19,47,48]. Morphogens allow different cells to express different proteins even though all cells within a morphology have identical genes and GRNs.

Besides morphogen gradients, another way to induce differential protein expression in real development is the asymmetric distribution of proteins between cells [49]. To model this, we distribute the concentrations of two non-morphogen TFs asymmetrically between cells at the initial 64-cell stage. We restrict one of these TFs to 32 cells left of the vertical centre line and the other to 32 cells below the morphology’s horizontal centre line. Thus, the cells in each of the four sectors in the spheroid each have unique concentrations of these two TFs (illustrated in Fig 1E), although whether this asymmetry persists over development depends on the gene regulatory network.

To visualise the model, cells are distinguished based on the tension or adhesion proteins they express by assigning each cell a state defined as a vector of Boolean values, where each Boolean value indicates whether a protein is expressed or not (Materials and methods). All cells with the same state are shown with the same colour on the CPM grid (Fig 1). Cell states are only used to visualise and analyse model outcomes and do not play any role in model dynamics.

To simulate the evolution of morphogenesis, we generated an initial population of 60 morphologies (Fig 1E), with each assigned a different randomly generated GRN. Each morphology develops on a separate CPM grid. We applied a genetic algorithm to select for shape complexity by measuring inward folding and deviation of the morphological shape from a circle at the end of the 12,000 DTS (Fig 1C and 1D; see Materials and methods). The 15 morphologies with the highest shape complexity reproduce four times to populate the next generation, and their GRNs undergo a single mutation with a probability of 50%. A mutation changes the regulatory effect of one TF on one gene, such as causing a TF to switch from inhibiting the expression of a gene to activating its expression (see Fig 1E). The GRNs used in our main set of simulations are comprised of nine transcription factors (including three morphogens), 15 adhesion proteins and two membrane tension proteins. Gene duplication and deletion do not occur. To broadly explore the types of morphogenesis our model evolves, we also run simulations where morphologies start as a rectangular initial shape with proteins asymmetrically distributed along the longest axis of the rectangle (S1 Fig), simulations where morphogen diffusivity mutates alongside GRN mutations (S1 Fig), simulations with different selection pressures (S2 Fig; see Materials and methods) and simulations with other numbers of genes (S3 Fig).

### Reproducibility is not an intrinsic property of complex morphogenesis

To investigate whether morphogenesis is intrinsically reproducible, we conducted 126 independent evolutionary simulations of our model starting from a circular initial condition. We ran each simulation for at least 2.5 × 10^3^ generations, which is usually sufficient to reach a plateau in fitness (S4 Fig). We then identified the fittest morphology (i.e., most complex shape) from the final generation (hereafter referred to as an “evolved morphology”) from each simulation. To ensure that we were analysing the reproducibility of complex shapes, we removed 36 evolved morphologies that did not reach an arbitrary threshold of complexity (our results are similar when a different threshold is used; S5 Fig). The 90 morphologies above this threshold each display a different shape (S4 Fig), with the evolution of two representative morphologies illustrated in Fig 1F and 1G, termed morphology-1 and morphology-2, respectively.

**Fig 2:**
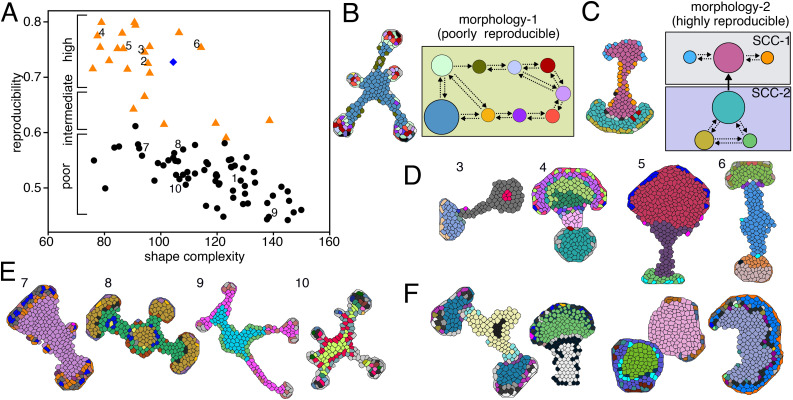
Both highly and poorly reproducible morphologies evolve in response to selection for shape complexity. **(A)** Reproducibility scores against shape complexity scores for the 90 morphologies that reached a threshold of shape complexity. Circles indicate morphologies with a single strongly connected component (SCC) (mean reproducibility = 52.0%, *n* = 65); filled triangles indicate morphologies with multiple SCCs with unidirectional transitions between them (mean reproducibility = 72.1%, *n* = 24). The blue diamond is a morphology with multiple SCCs without unidirectional transitions (S6 Fig). Numbered arrows refer to the morphologies in panels B, C, D, and E. **(B, C)** Simplified cell state spaces for (B) morphology-1 and (C) morphology-2. Node colours correspond to cell state colours depicted in the morphologies to the left each of cell state space. Arrows are cell-state transitions. Cell states are partitioned into strongly connected components (SCCs, coloured boxes). Node sizes depict cell state frequency over all of development; node colours correspond to cell states from morphology-1 and morphology-2, respectively (shown to the left of each state space). See S7 Fig for the state spaces without pruning of nodes and edges. **(D)** Four morphologies that are highly reproducible. **(E)** Four morphologies that are poorly reproducible. **(F)** Four highly reproducible morphologies from simulations where morphologies evolved with morphogens mutating (for more information see S1 Fig).

We examined whether the 90 evolved morphologies with complex shapes display reproducible morphogenesis by repeatedly simulating their development with different pixel copy orders (60 replicates per morphology, hereafter referred to as developmental replicates). To quantify reproducibility, we measured a “reproducibility score”, which indicates how geometrically similar the morphologies of replicates are to each other (see Materials and methods). The reproducibility score depends on the geometry, size and time taken to generate the morphology but is invariant to reflection, rotation and translation of the morphology. The distribution of reproducibility scores across all 90 evolved morphologies is bimodal (bimodality coefficient = 0.69, Fig 2A), with 19 morphologies in the upper mode, which we term highly reproducible (including morphology-2; Fig 2D shows four other examples), 65 morphologies in the lower mode, which we term poorly reproducible (including morphology-1; Fig 2E shows four other examples), and six that are between the two modes, which we term intermediately reproducible (see S8 Fig for information about intermediately reproducible morphologies). The morphologies in the lower mode also tend to have higher variation in shape complexity than those in the higher mode, supporting our assertion that they are less reproducible (S9 Fig). The bimodal distribution implies that evolved morphologies consist of a mixture of two populations with distinct properties.

### Highly and poorly reproducible morphologies have distinct cell-state transition dynamics

To understand how differences in reproducibility between morphologies arise, we investigated how these differences covary with shape complexity or the number and spatial distribution of cell states. While we found that differences in reproducibility could not be explained by shape complexity (see S1 Appendix, S10 Fig) or the number of cell states (S11 Fig), we noticed a stark difference in the spatial distribution of cell states between highly and poorly reproducible morphologies, as depicted by the distribution of cell colours in the morphologies shown in Fig 2. Specifically, in highly reproducible morphologies, each cell state is localised to a specific morphological domain. By contrast, in poorly reproducible morphologies, cell states are unlocalised, reappearing all over each morphology.

We asked whether differences in spatial distributions of cell states in highly and poorly reproducible morphologies are due to differences in cell-state transition dynamics, i.e., how and which cells transition between states. To answer this question, we recorded all cell-state transitions of all cells in each evolved morphology to generate a “cell state space” for each morphology, which is defined as a graph consisting of nodes representing cell states and edges representing all possible transitions between cell states (see Materials and methods). Since each cell state space contains numerous nodes and edges, we simplified the state spaces in two steps. First, we pruned infrequently observed cell states and cell-state transitions. Second, we split the cell state space into strongly connected components (SCCs), where an SCC is a set of cell states for which there exists a transition pathway (direct or indirect) from any cell state to any other cell state within that set. We found that almost all (64 out of 65) poorly reproducible morphologies had only a single SCC, as illustrated for morphology-1 in Fig 2C. In contrast, all 19 highly reproducible morphologies and most (five out of six) intermediately reproducible morphologies had cell state spaces containing multiple SCCs, as illustrated for morphology-2 in Fig 2B. Moreover, most (24 out of the 25) morphologies with multiple SCCs had at least one SCC that unidirectionally transitioned to another SCC (Fig 2A; see S6 Fig for details about morphologies without unidirectional SCC transitions). The difference in reproducibility between morphologies with a single SCC and those with multiple SCCs is statistically significant (*p* < 10^−12^, two-tailed t-test), suggesting that the presence of multiple SCCs is involved in morphogenetic reproducibility.

**Fig 3:**
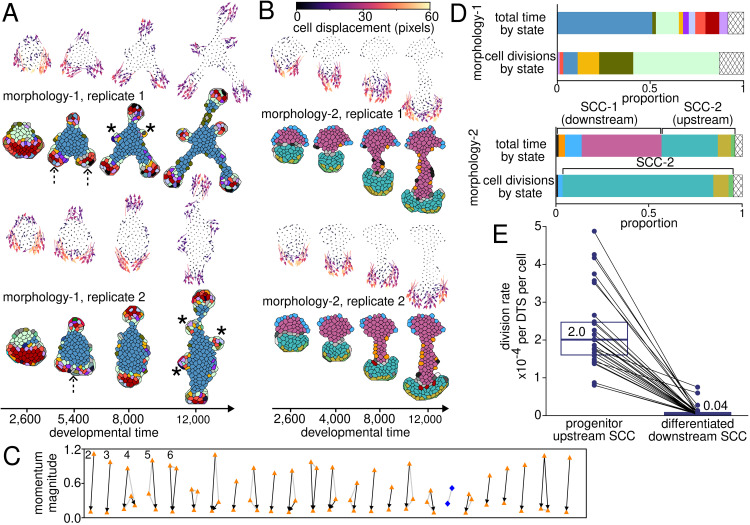
Highly reproducible morphologies have moving and dividing cells that undergo unidirectional transitions to non-moving and non-dividing cells. **(A, B)** Two developmental replicates of morphology-1 (A) and morphology-2 (B) are depicted after 2,600, 4,000, 8,000 and 12,000 DTS, showing a difference in their reproducibility. Dashed arrows in (B) indicate the presence (replicate-1) or absence (replicate-2) of a bifurcation in collective cell motion; asterisks indicate protrusions. Vector plots show the displacement of the centre of mass of each cell during 2,000 DTS at each respective time point, with colours indicating magnitude (the lighter, the larger). **(C)** Average cell momentum magnitude for each SCC from the 25 morphologies with multiple SCCs. Momentum is the distance travelled by a cell per DTS multiplied by its size in pixels. Black arrows indicate unidirectional transitions between SCCs. Grey lines connect SCCs from the same morphology that do not have unidirectional SCC transitions between each other. Filled orange triangles are SCCs from morphologies that have unidirectional SCC transitions. All transitory SCCs are excluded (see Materials and methods for information about transitory SCCs). Blue diamonds are SCCs from the highly reproducible morphology that does not have unidirectional SCC transitions. The numbered morphologies correspond to those from Fig 2D. **(D)** Stacked bar charts showing the proportion of developmental time spent in each cell state and the proportion of cell divisions undergone by each state across all cells during developmental replicate-1 of morphology-1 and development replicate-1 of morphology-2. Diagonal lattices are pruned states. **(E)** The rate at which cells divide per developmental time when their state belongs to an upstream SCC (left) or a downstream SCC (right). Each data point represents an SCC from **(C)**. Black lines connect upstream SCCs to their counterpart downstream SCCs. Boxes show medians and interquartile ranges (IQR); the downstream SCC box is tiny because most division rates are either very low or 0. Numbers on top of the box plots are median cell division rates.

To investigate why there is a relationship between the number of SCCs and reproducibility, we examined the relationship between cell states and cell motions, given that cell motion drives morphogenesis and differences in cell motions across developmental replicates drive reproducibility. To examine cell motions, we visualised the velocity of every cell at multiple time points during the development of evolved morphologies across multiple developmental replicates (here using morphology-1 and morphology-2 as examples). The velocity plots reveal two ways in which morphology-1 (one SCC) undergoes morphogenesis. The first way is “bifurcations”, in which a cluster of moving cells splits into two clusters (Fig 3A and S2 Movie). We hypothesised that bifurcations are induced by the transition of moving cells to stationary cells at the cluster tip. While observing cell trajectories suggested that these transitions cause bifurcations, the complex interactions between morphogen signalling, adhesion, and cell division make it difficult to isolate causal mechanisms through observation alone. Thus, we tested if moving-to-stationary transitions cause bifurcations by artificially converting the states of three moving cells to stationary states at the cluster tip. We performed this conversion by extracting the protein and morphogen profiles of an arbitrary stationary cell with the same morphology, and overwriting the states of three adjacent moving cells with these extracted profiles. The result shows that this conversion induced a bifurcation (S12 Fig), supporting the hypothesis. In the two developmental replicates shown in Fig 3B, a bifurcation occurs in replicate-1 but not in replicate-2, indicating bifurcations are prone to stochasticity.

The second way in morphology-1 (one SCC) undergoes morphogenesis is “protrusions”, where a new cluster of moving cells forms (Fig 3A). We hypothesised that protrusions are induced by the onset of collective motion from previously stationary cells. To test this, we converted the states of three stationary cells to moving cell states using the same conversion protocol described previously. The result shows that this conversion induced a protrusion (S12 Fig), supporting the hypothesis. In the two developmental replicates shown in Fig 3B, two cell groups protrude in replicate-1 around 8,000 DTS, whereas three cell groups protrude in replicate-2 around 12,000 DTS in different locations from replicate-1. This result indicates that protrusions are also prone to stochasticity.

Together, these results indicate that morphogenesis in morphology-1 is driven by noise-prone transitions between moving and stationary states, which reduces reproducibility. Because all states belong to the same SCC, these transitions are possible and can be noise-prone. Evolution likely selects for more noise-prone transitions because it promotes morphogenesis, thereby increasing shape complexity and overall fitness. These results are not limited to morphology-1: noisy transitions between moving and stationary states appear to drive morphogenesis in most of the poorly reproducible morphologies we evolved, even for those with vastly different mechanics, such as “finger” formation via cell death (S6 Fig, S5 Movie). Consequently, these noisy transitions are a fundamental cause of poor reproducibility in our model.

We hypothesised that moving and stationary states are partitioned into separate SCCs in highly reproducible morphologies, thereby preventing noisy transitions between them. We first tested this hypothesis on highly reproducible morphology-2 (two SCCs). The velocity plots for morphology-2 reveal that cells in states belonging to one of its two SCCs collectively move downwards and radiate slightly outwards, like a travelling wave (Fig 3A; S1 Movie). Cells in states belonging to the other SCC show little motion. These position-dependent collective cell motions generate a “cap” of moving cells and a “stalk” of stationary cells forming in the wake of the moving cap (Fig 3A). These observations suggest that moving and stationary states are not only dynamically partitioned into distinct SCCs as we hypothesised, but also spatially partitioned into distinct domains in morphology-1.

To confirm that different SCCs have different cell-motion properties across all highly reproducible morphologies, we measured the momentum of cells in each SCC (Materials and methods). The result shows a significant disparity (mean 6.7-fold difference) in the average magnitude of cell momentum between SCCs across all highly reproducible morphologies (Fig 3C), indicating that moving and stationary states are indeed partitioned into separate SCCs. Moreover, when there are unidirectional transitions between SCCs (24 out of 25), the transitions are always from high momentum upstream SCCs to low momentum downstream SCCs (black arrows in Fig 3C). These results indicate that noisy transitions from stationary to moving cells (for instance, those that lead to protrusions in morphology-1) do not occur in highly reproducible morphologies because stationary cell states cannot transition to moving cell states, which is likely a contributing factor towards their higher reproducibility.

We wondered, mechanistically, what causes the difference in mobility between cell states in different SCCs. To answer this, we compared the cell-cell adhesion energy among cells from upstream SCCs to the cell-cell adhesion energy among cells from downstream SCCs, since cell-cell adhesion energies are the key component of cell mobility in our model. We found that the cell-cell adhesion energy was much lower among those from upstream SCCs (mean 1.39) than those from downstream SCCs (mean 3.51), with no overlap between groups (S13 Fig). This difference in cell-cell adhesion energies means that cells in upstream SCCs behave more fluid-like, whereas cells in downstream SCCs behave more solid-like.

Since moving cells in upstream SCCs do not disappear despite transitioning to stationary cells in downstream SCCs, we thought moving cells might be dividing rapidly. To test this, we compared how often cells in moving versus stationary SCCs divide across all morphologies with multiple SCCs (Fig 3D and 3E). We found that cells in moving SCCs divide a median of 49.6 times more frequently than those in stationary SCCs. Together, these asymmetries in cell motion, adhesion and division indicate that highly reproducible morphologies establish a “morphogenetic division of labour”, whereby domains of moving, dividing cells “shape” morphologies whilst stationary cells “maintain” the morphologies shaped by moving cells.

Since our model has specific initial conditions, genetic parameters, and selection pressures, we sought to confirm that the complex, reproducible morphogenesis driven by morphogenetic division of labour is robust to these variables, rather than evolving as an artefact of our specific simulation setup. To this end, we ran simulations of our model with different initial shapes (S1 Fig), simulations where morphogen diffusivity can mutate (S1 Fig), simulations with different selection pressures (S2 Fig) and simulations with different numbers of genes (S3 Fig). We found that morphogenetic divisions of labour that confer complex yet reproducible morphogenesis evolved in all of these kinds of simulations. Moreover, morphogenetic divisions of labour evolved in 80–90% of simulations when we modified the selection criterion to indirectly select morphogenesis that undergoes directional motion (which indirectly selects for reproducibility; Materials and methods and S2 Fig), indicating that morphogenetic divisions of labour are easy to evolve from different initial GRNs. Given that these morphogenetic divisions of labour resemble progenitor-cell systems found in animal development [2], where progenitor cell states irreversibly differentiate into one or a few differentiated cell types, we hereafter define upstream SCCs as progenitor-cell types and downstream SCCs as differentiated-cell types. We refer to unidirectional transitions from progenitor to differentiated as cell differentiation. We use these terms to denote the hierarchical and functional roles of cell states within our model, acknowledging that they represent a simplified abstraction of more complex regulatory and epigenetic states found in real animal cells.

### Regulated cell-motion transitions at domain boundaries enables complex yet reproducible morphogenesis

**Fig 4:**
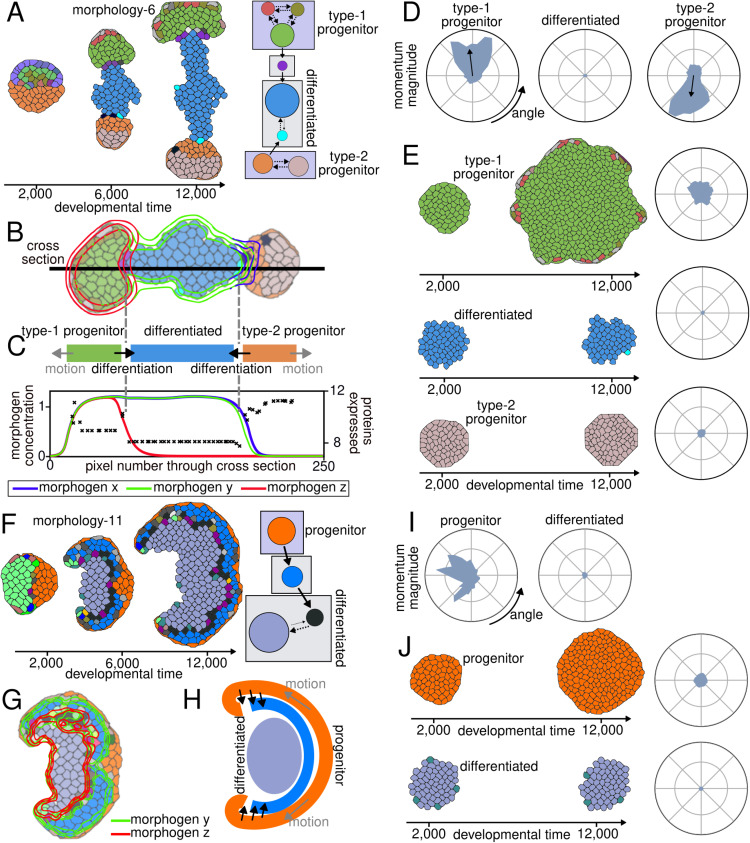
Mechanisms underlying progenitor-cell differentiation and motion. **(A)** Development and cell state space of morphology-6, showing two progenitor-cell types, one differentiated-cell type, and a transitory SCC (see Materials and methods for information about transitory SCCs). The morphology is shown after 2,000, 6,000 and 12,000 DTS. **(B)** Contours showing the concentrations of the three morphogens (*x*, *y* and *z*) overlaid on morphology-6 after 9,000 DTS. Each contour joins points of equal concentration of the same morphogen. **(C)** Schematic depicting type-1, type-2 and differentiated cell domains from **(B)**, with progenitor-cell motion (grey arrows) and differentiation (black arrows) indicated. Vertical dashed lines indicate cell-type boundaries. Below, morphogen concentrations along the cross-section line are plotted, along with the sums of cell protein concentrations for cells along the cross-section (each cross is one cell). **(D)** Polar plots of momentum magnitude by angle of motion for each cell type summed over all cells over the 12,000 DTS of morphology-6 development. **(E)** Development of type-1, type-2 and differentiated cells in isolation. Polar plots show distributions of cell momentum as in **(D)**. **(F)** Development and cell state space of morphology-11, showing a progenitor-cell type, a differentiated cell-type and a transitory SCC. The morphology is shown after 2,000, 6,000 and 12,000 DTS and its state space. **(G)** Contours showing the concentrations of morphogens *y* (green) and *z* (orange, morphogen *x* is hidden for visibility) overlaid on morphology-11 after 8,000 DTS. **(H)** A schematic depicting progenitor and differentiated cell domains for morphology-11, with progenitor-cell motion (grey arrows) and differentiation (black arrows) indicated. The combined presence of morphogens *y* and *z* induces differentiation of progenitor cells. **(I)** Polar plots of momentum magnitude by angle of motion for progenitor and differentiated cell types summed over all cells over the 12,000 DTS of morphology-11 development shown in **(F)**. **(J)** Development of progenitor and differentiated cells from morphology-11 in isolation. Polar plots show distributions of cell momentum as in **(I)**.

We wondered how morphogenetic divisions of labour increase morphogenetic reproducibility despite frequent cell differentiation from moving states (progenitor cells) to stationary states (differentiated cells), which cause poor reproducibility in evolved morphologies with only a single SCC, because these transitions are prone to noise. We noticed that this differentiation occurs at a steady rate and only at the boundaries between domains of progenitor and differentiated cell types, suggesting that differentiation does not reduce reproducibility because it is tightly regulated. For example, in morphology-6, which has two progenitor cell types denoted type-1 and type-2 (Fig 4A), progenitor cells initially differentiate exclusively at the boundary between the type-1 and type-2 progenitor cells and, subsequently, exclusively at the boundary between progenitor cells and differentiated cells (S3 Movie). Both progenitor-cell domains are present very early in morphology-6 development (i.e., just after the initial 64-cell stage). Whether a cell starts at type-1 or type-2 depends on the concentration of the TF distributed asymmetrically across the horizontal centre line. Since type-1 and type-2 stem cells start in distinct domains, the spatial layout of progenitor and differentiated cells ends up mirroring the topology of the cell state space, with differentiated cells consistently forming between the progenitor-cell domains (Fig 4A; see S7 Fig for other examples).

Boundary-localised differentiation suggests that progenitor-cell differentiation is spatially regulated. To determine how this regulation is achieved, we focused on morphology-6 as it has two separate examples of progenitor-cell differentiation. We hypothesised that boundary-localised differentiation is caused by morphogen-mediated interactions between progenitor and differentiated cells, as morphogens provide a means to spatially regulate gene expression. To test this, we made a contour plot of morphogen concentrations for morphology-6 (Fig 4B). The plot shows that the concentrations of different morphogens abruptly change at the boundaries between progenitor and differentiated cells (dashed lines in Fig 4B). To determine whether gene expression responds to these changes in morphogen concentrations, we plotted the sum of protein concentrations (excluding morphogens) for cells along a cross-section of morphology-6 (Fig 4C). The plot shows that this sum changes abruptly wherever morphogen concentrations change. These results suggest that a specific morphogen profile induces differentiation, with differentiated cells producing this profile and thus localising differentiation to the boundary between progenitor and differentiated cells. To directly test this, we isolated each progenitor-cell type from the other two cell types, thereby removing the effect of differentiated cells on morphogen profiles. We isolated cell types by creating morphologies in which all cells in the initial spheroid were set to the state that is most frequently observed for each of the progenitor-cell types (e.g., the green cell state shown in Fig 4A for type-1 progenitor cells). We developed these morphologies for 12,000 DTS, and found that neither type-1 nor type-2 progenitor-cell types differentiate (Fig 4E). To test the robustness of our results, we repeated the above analyses on morphology-11, which undergoes a different kind of morphogenesis from morphology-6—progenitor cells spread around the surface of the morphology before differentiating like epiboly [50] (Fig 4F, S4 Movie). We found that morphology-6 progenitor cells differentiate in response to the combined exposure to two types of morphogens (Fig 4G and 4H), and that progenitor cells do not differentiate when isolated from differentiated cells (Fig 4J). These results support the hypothesis that differentiated cells induce progenitor-cell differentiation, thus localising differentiation to the boundaries between cell types.

We next asked whether the above findings—that differentiated cells induce progenitor-cell differentiation—are generalisable to all morphologies with progenitor-cell differentiation. To answer this, we isolated each of the 30 progenitor-cell types from the 24 morphologies with progenitor-cell differentiation from all other cell types and tested whether the isolated progenitor cells differentiate through the same method as described in the previous paragraph. We found that the great majority (26 out of 30) do not differentiate when isolated from other cell types (S14 Fig shows why the four exceptions do not counter our hypothesis). This result, along with the observation that every progenitor-cell type always differentiates at the boundary shared with differentiated cells (Figs 3A and 4), indicates that differentiated cells induce progenitor-cell differentiation.

**Fig 5:**
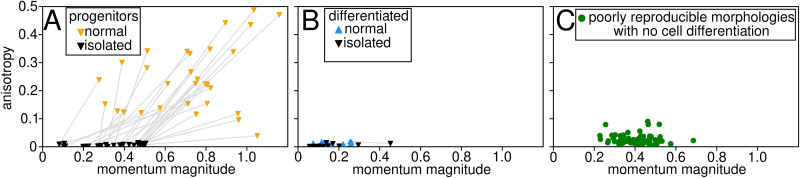
Interactions between cell types drive anisotropic progenitor-cell motion. **(A)** Motion anisotropy of progenitor cells within morphologies (orange) and isolated (black) plotted against average cell momentum magnitude (*n* = 30 cell types). Grey lines connect a progenitor-cell type within a morphology to that progenitor-cell type in isolation. **(B)** The same as **(A)**, but for differentiated cells (*n* = 26 cell types). **(C)** The same as **(A)**, but for cells from morphologies with only one SCC (*n* = 65).

The spatial consistency of progenitor-cell differentiation is crucial but not sufficient for complex yet reproducible morphogenesis. The other critical factor is the motion of progenitor cells in a consistent direction, as this motion creates the complex shapes that are conserved across developmental replicates. Given that progenitor-cell differentiation is induced by differentiated cells, we hypothesised that the directionality of progenitor-cell motion is also induced by differentiated cells. To test this, we determined how progenitor cells’ motion depends on the presence or absence of differentiated cells by measuring the magnitude of progenitor cells’ momentum as a function of the angle of momentum, either in normal development (differentiated cells present) or when each progenitor-cell type is isolated (differentiated cells absent). The result shows that cell momentum is strongly directional (i.e., anisotropic) in normal development, whereas it is radially symmetrically distributed (i.e., isotropic) when cells are isolated (Fig 4 shows results for morphology-6 and morphology-11; see S14 Fig for other morphologies). We quantified this difference in anisotropy by calculating the ratio of the variance in momentum magnitude across angle to the mean momentum magnitude across angle (Materials and methods). We found that anisotropy of progenitor-cell motion decreased an average of 65-fold across the 30 progenitor-cell types when progenitor cells were isolated (Fig 5). To understand mechanically how this anisotropic motion occurs, we analysed the expression of adhesion proteins across morphologies with progenitor-cell differentiation, given that our model is primarily adhesion-based. We found that across all morphologies with progenitor-cell differentiation, progenitor and differentiated cells consistently differed in either their adhesion to one another or their adhesion to the medium (S13 Fig). However, the exact mechanism relating differential adhesion to anisotropic motion depends on the morphology (e.g., morphology-6 vs morphology-11 in Fig 4). Together, these results indicate that interactions between domains of progenitor and differentiated cells (differentiation, signalling, adhesion) transform independently “simple” domains, which are trivially reproducible, into complex ones without sacrificing reproducibility.

## Discussion

We investigated whether reproducible morphogenesis evolves when it is not directly selected in a computational model of development. We found that the evolved, complex morphologies displayed a bimodal distribution in reproducibility scores, i.e., highly and poorly reproducible morphogenesis emerged as a by-product of evolution. Our results reveal that highly reproducible morphogenesis adheres to two principles that are violated in poorly reproducible morphogenesis. First, moving and stationary cell states are segregated into distinct domains (e.g., tissues). Second, transitions between these states, if at all, occur unidirectionally at domain boundaries. These principles are consistent with previous theoretical and experimental works, which showed that high stability of domain boundaries is important for morphogenetic reproducibility [26,51]. Moreover, these two principles are broadly consistent with real animal development. Consistent with the first principle, animal cells tend to be spatially segregated into fluid-like collectives or solid-like collectives [52]. Examples of this spatial segregation include motile “tip” cells from “stalk” cells during branching morphogenesis [53], fluid-like mesenchymal cell condensates from solid-like mesenchymal cells during elongation morphogenesis [54], and unjammed mesoderm from jammed mesoderm in tail elongation [55]. However, exceptions to this spatial segregation exist, such as the “salt-and-pepper” cell sorting in the mouse blastocyst, where motile endoderm cells crawl through stationary epiblast neighbours [56]. Consistent with the second principle, transitions between fluid-like and solid-like cell states typically occur at boundaries between domains of moving and stationary cells, such as mesenchymal-to-epithelial transitions during nephrogenesis [57] and somitogenesis [9]. Thus, the spatial segregation of moving and stationary collectives with boundary-restricted transitions between them could be a fundamental strategy for ensuring reproducible morphogenesis.

Our evolutionary simulations show that the most common way to achieve highly reproducible yet complex morphogenesis is a morphogenetic division of labour, where domains of mobile, dividing progenitor cells “shape” morphologies and irreversibly differentiate into domains of stationary, non-dividing cells that “maintain” these morphologies. This labour division allows differentiated cells to serve as anchor points that establish the locations of progenitor-cell differentiation and directions of progenitor-cell motion via morphogen gradients and differential cell adhesion. Some morphologies that evolved with this labour division, such as morphology-11 (Fig 4F), resemble epiboly observed in systems like zebrafish gastrulation. In the zebrafish, cells undergo epiboly by spreading over the surface of the yolk before differentiating into a more solid-like periderm [50], similar to the spreading and differentiation of progenitor cells in morphology-11. Other morphologies that evolved, such as morphology-2 and -6 (Figs 3B and 4A), resemble elongated and bulge-like sub-structures in real animal development, such as intestinal villi [54,58], lung alveoli [53,59], mammary gland buds [60] and embryonic tail elongation [9]. For example, in gut villus elongation, high surface tension, fluid-like mesenchymal tissue progressively elongates the villus before differentiating into solid-like mesenchymal tissue [54]. Similarly, morphology-2 and -6, which progressively elongate, have progenitor cell domains with higher surface tension and fluidity than differentiated cells (S13 Fig).

One feature of the sub-structures like lung alveoli and mammary gland buds that morphogenesis in our model does not recreate is iterative branching. Our evolved morphologies undergo a maximum of one branching event during morphogenesis (S6 Fig, S6 Movie), likely because the set of cell properties we implemented is insufficient for tissues to undergo iterative branching. We hypothesise that iterative branching could be achieved by integrating morphogenetic division of labour with known mechanisms for branch point specification, such as localised inhibition of proliferation at the branching point (e.g., in the trachea [61]) or reciprocal signalling with the surrounding mesenchyme (e.g., in the kidney [57]).

A limitation of our current model is the diversity of morphogenesis it can evolve. We observed at least seven classes of morphogenetic dynamics in our model (see Figs 3A and 3B and 4 and S6 and S8). Both highly and poorly reproducible morphologies exhibited several kinds of dynamics, and we did not observe any difference in shape diversity between highly and poorly reproducible morphologies (S10 Fig). However, the diversity of morphogenesis in our model is much lower than observed across animal tissues. Diversity is lower than in animal tissues due to three aspects of its design. First, we implemented a set of cell properties deliberately constrained to those universally present in animal development: cell-cell adhesion, cortical tension, cell division, and gene regulation. We chose these properties to ensure the reproducibility-conferring principles we discovered would broadly apply to animal morphogenesis. However, this implementation limits the kinds of morphogenesis that can evolve to those driven by these cell properties. For instance, the absence of cell polarisation in our model is the likely reason we do not observe invaginations, since invaginations require polarisation [62,63]. Second, we simulated our model in 2D rather than 3D due to computational limitations. Simulating in 2D prevents the morphogenesis of 3D structures, such as tubes. Third, we only tried two evolutionary selection pressures in our model (shape complexity and directional motion). In contrast, real development is shaped by numerous and varied selection pressures acting on large populations over longer timescales. We expect that introducing more diverse selection pressures and ecologies would increase the morphogenetic diversity our model produces. That our model does not replicate the full diversity of animal tissues does not invalidate the reproducibility-conferring processes we discovered. However, further research is needed to determine whether these processes apply only to the limited kinds of morphogenesis driven by the cell properties we implemented (e.g., those involving cell divisions, differential adhesion and cortical tension) or apply to other kinds of morphogenesis.

Our findings indicate that cell differentiation does more than specify cell types; it can drive morphogenesis and even possibly tissue regeneration. This role of cell differentiation may be important for *in vitro* tissue development, such as in organoid cultures, where programming accurate and reproducible morphogenesis remains a major challenge in synthetic biology [64,65]. Our model predicts that organoid morphogenesis can be made more reproducible by establishing scaffolds composed of solid-like, stationary cells generated through cell differentiation (S15 Fig). Organoids must generate their own structural support because they develop in isolation from a cluster of progenitor cells, lacking the natural physical constraints found *in vivo*. By inducing specific cells to act as scaffolds and localised sources of morphogens, the behaviour of progenitor cells could be guided to more reproducible morphogenesis. Intestinal organoids and mammalian gastruloids are two systems that could benefit from this approach [66,67]. These systems begin as progenitor cell collectives that progressively differentiate as they undergo morphogenesis. Our modelling predicts that controlling when and where progenitor cells begin to differentiate will improve reproducibility. However, the differentiated cells in these organoid systems may require genetic modifications to serve as chemical and physical scaffolds, as in our model. Alternatively, these organoid systems may require entirely different kinds of scaffolds. Future work could bridge the gap by modelling how different kinds of scaffolds contribute to the reproducibility of organoid morphogenesis.

In summary, our results suggest that cell differentiation, in addition to producing specialised cell types, can underpin complex yet reproducible morphogenesis. Our findings open the possibility that cell differentiation during the development of some animal tissues evolved for a morphogenetic purpose, with specialised cell types emerging as a later exaptation.

## Materials and methods

### Cellular potts model

Our model extends a Cellular Potts Model (CPM) introduced by Hogeweg (2000) [29] by adapting an implementation of the Tissue Simulation Toolkit [68,69]. The CPM dynamics are driven by pixel copying, where repeated random sampling of pixels on grid determines the location of these copies. For each chosen pixel, a random neighbouring pixel from its Moore neighbourhood is selected as the recipient of the pixel copy. Whether the pixel copy is accepted depends on its effect on the system’s energy, denoted as *H*, represented by


$$\begin{array}{c} H=\sum_{i,j}J_{ij}+\sum_{i,m}J_{im}+\lambda_{V}\sum_{\sigma }(\upsilon_{\sigma }-V_{\sigma }{)}^{2}+\sum_{\sigma }\lambda_{L}^{\sigma }(l_{\sigma }-L_{\sigma }{)}^{2}. \end{array}$$ (1)

Here, *H* encompasses the total surface energy accumulated from cell-cell adhesion (*J*_*ij*_) and cell-medium adhesion (*J*_*im*_), which are both determined dynamically as functions of protein concentrations (explained later). The index *i* is of pixels at cell boundaries. The index *j* is of pixels neighbouring *i* that are occupied by a different cell from *i*. The index *m* is of pixels neighbouring *i* that are occupied by the medium. Every pixel on the grid has an associated value, σ, that represents either the cell that occupies that pixel (σ≥1) or the medium (σ=0). Each cell σ (σ≥1) with current size $\upsilon_{\sigma }$ (in pixels) is constrained to size $V_{\sigma }$ with parameter $\lambda_{V}$ ($\lambda_{V}=0.5$ for all simulations). The longest axis $l_{\sigma }$ (in pixels) of each cell is constrained to length $L_{\sigma }$ with $\lambda_{L}^{\sigma }$. $V_{\sigma }$, $L_{\sigma }$ and $\lambda_{L}^{\sigma }$ are determined dynamically (explained later).

If a pixel copy attempt increases *H*, it is accepted with probability $e^{-\frac{\Delta H}{T}}$, where ΔH is the change in *H* made by a pixel copy, and *T* is a temperature-like parameter that we arbitrarily fixed to *T* = 3. Otherwise (ΔH≤0), the pixel copy attempt is always accepted. One developmental time step (DTS) is complete when the number of pixel copy attempts equals the number of pixels on the grid.

### Development

A morphology starts as a single cell that undergoes six divisions equally spaced in time over the first 300 DTS. During the first 1,500 DTS (referred to as an equilibration phase), $J_{ij}=30$ and $J_{im}=40$ for all cells. After the equilibration phase, *J*_*ij*_ and *J*_*im*_ are determined by protein concentrations in the cells (described later). At the beginning of the equilibration phase, $V_{\sigma }$ is set to the initial size of each of the 64 cells and does not change throughout the equilibration phase. After the equilibration phase, cell growth and shrinkage can occur through modulation of the target cell size, $V_{\sigma }$. $V_{\sigma }$ increases when a cell is stretched, and decreases when a cell is squeezed, as follows. When $\upsilon_{\sigma }\geq V_{\sigma }+3$, $V_{\sigma }$ is updated such that $V_{\sigma }=\upsilon_{\sigma }$. When $\upsilon_{\sigma }\leq V_{\sigma }-16$, $V_{\sigma }$ is updated such that $V_{\sigma }=\upsilon_{\sigma }$. When the cell volume reaches or exceeds a threshold (i.e., $\upsilon_{\sigma }\geq 100$), the cell undergoes division perpendicular to its longest axis. One daughter cell retains the same index σ as the mother cell, while the other daughter cell is assigned a new index $\sigma^{\prime }$. After division, the target area of the daughter cells’ are $V_{\sigma }=\upsilon_{\sigma }$ and $V_{\sigma^{\prime }}=\upsilon_{\sigma^{\prime }}$. Protein concentrations remain unchanged upon cell division. If $\upsilon_{\sigma }=0$, the cell dies.

### Gene regulatory network and morphogens

We model protein concentration dynamics using a system of ordinary differential equations that correspond to the reaction-only component of the Reinitz and Sharp model [70]. In each cell σ, there are *N*_*genes*_ genes, indexed by *p*, that each encode a protein whose intracellular concentration is denoted by $x_{p}^{\sigma }$. With the exception of


$$\begin{array}{c} p\in \{1,\ldots ,N_{morph}\} \end{array}$$

(which are morphogens, described later), the following equation determines the change in $x_{p}^{\sigma }$ over time *t*:


$$\begin{array}{c} \frac{dx_{p}^{\sigma }}{dt}=\frac{a}{1+e^{-\beta f_{p}(x)}}-bx_{p}^{\sigma } \end{array}$$ (2)

with one unit of *t* being one DTS. The first term on the right-hand side represents the increase in $x_{p}^{\sigma }$ due to gene expression, and is a sigmoidal function that depends on transcription factor regulation, *f*_*p*_(*x*) (described subsequently), with a maximum production rate *a* and large β (=20). The second term represents protein decay with rate *b*. We set *a* = *b* for all *p*. This ensures that when a gene is activated (*f*_*p*_(*x*) > 0), the production term dominates and $x_{p}^{\sigma }\to_{\;\;}^{\;\;}1$. Conversely, when a gene is inhibited (*f*_*p*_(*x*) < 0), the production term is negligible compared to the decay term and $x_{p}^{\sigma }\to_{\;\;}^{\;\;}0$. Thus, the expression dynamics are a switch-like response where genes transition between “on” or “off”, i.e., $x_{p}^{\sigma }$ equilibrates at 1 if the gene is constantly expressed or 0 if it is constantly not expressed. We assume that the timescale of gene expression is slower than the timescale of CPM dynamics. Thus, we set *a* and *b* to small values (specifically, 6.25 × 10^−3^). Numerical integration of Equation 2 occurs with $\Delta t_{1}=40$ via the Euler method (we use a subscript because Equation 4, described subsequently, is numerically integrated at a different interval). We chose this value of $\Delta t_{1}$ instead of a smaller value to improve computational speed. The value of $\Delta t_{1}$ can be large because the rate parameters *a* and *b* are very small. The initial concentrations are $x_{p}^{\sigma }=1$ for transcription factors (except for the two that are asymmetrically distributed, which are either $x_{p}^{\sigma }=1$ or $x_{p}^{\sigma }=0$ depending on σ at the four-cell stage), and $x_{p}^{\sigma }=0$ for all other proteins (described later). Integration of Equation 2 begins after the four-cell stage is reached (100 DTS), which is when the asymmetric distribution of transcription factors (TFs) occurs.

Function *f*_*p*_(*x*) in Equation 2 sums the regulatory effects of *N*_*TF*_ TFs (including morphogens) as follows:


$$\begin{array}{c} f_{p}(x)=\left[\sum_{p^{\prime }}^{n}Z_{pp^{\prime }}x_{p^{\prime }}^{\sigma }\right]+\theta \end{array}$$ (3)

where $Z_{pp^{\prime }}$ is the regulatory effect of TF encoded by gene $p^{\prime }$ on the expression of gene *p* ($Z_{pp^{\prime }}\in \{0,\pm 1,\pm 2\}$). The TF encoded by $p^{\prime }$ activates the expression of *p* if $Z_{pp^{\prime }}>0$, inhibits if $Z_{pp^{\prime }}<0$ and has no effect if $Z_{pp^{\prime }}=0$. The parameter θ sets the base level of gene expression. θ=−0.3 for all simulations, so protein concentrations equilibrate at 0 when they are not regulated by any TFs.

To model cell-cell signalling, the first *N*_*morph*_ ($p=1,\ldots ,N_{morph}$) of the *N*_*TF*_ TFs are morphogens. These morphogens diffuse between cells and into the surrounding medium. The concentration of morphogen *p* on pixel *i* on the grid, $x_{p}^{i}$, is determined by the following coupled ODE:


$$\begin{array}{c} \frac{dx_{p}^{i}}{dt}=D_{p}\nabla^{2}x_{p}^{i}+\omega_{p}H\left(\sigma -1\right)x_{p_{sig}}^{\sigma }-\eta x_{p}^{i} \end{array}$$ (4)

where *D* is a diffusion constant, ω is a production rate, η is a decay rate and H(σ−1) is the Heaviside step function that evaluates to one if σ≥1 at pixel *i* (the pixel is occupied by a cell) and zero if σ=0 (the pixel is medium). The concentration $x_{p_{sig}}^{\sigma }$ represents a signalling protein that activates the expression of morphogen *p*. The concentration of signalling proteins is determined by Equation 2. The operator $\nabla^{2}x_{p}^{i}$ is the Laplacian acting on $x_{p}^{i}$, which, in this context, is $x_{p}^{i}$ subtracted from the average of $x_{p}^{i}$ in its von Neumann neighbourhood (i.e., four nearest-neighbouring pixels) divided by a space step (*dx*)^2^, with *dx* = 1/250 (250 is the length of the square grid in pixels). Numerical integration for Equation 4 occurs with $\Delta t_{2}=1$. Equation 4 is subject to the initial condition $x_{p}^{i}=0$ for all *i* and *p*. The pixels at the boundary of the grid are subject to $x_{p}^{i}=0$ for all *t* and *p*. The constants $D_{p}=8\times 10^{-7}$, $\omega_{p}=2.4\times 10^{-3}$ and $\eta =2\times 10^{-3}$ for all *p* were used for the 126 simulations outlined in the main text. We chose these values for two reasons. The first is so that the maximum concentration is approximately 1 and thus similar to the concentrations of TFs per Equation 2. The second is so that the characteristic diffusion length, $\sqrt{D_{p}/\eta }/dx$, is similar to that of a paracrine morphogen, such as Wnts, which signal only to nearby cells [48,71]. This characteristic diffusion length is five pixels, which is approximately the diameter of a cell. Since $x_{p}^{i}$ regulates genes in each cell σ, we average $x_{p}^{i}$ over all *i* with the value σ to obtain $x_{p}^{\sigma }$. However, we also ran 44 simulations where *D*_*p*_ and $\omega_{p}$ mutate (S1 Fig and Materials and methods Section Evolution), resulting in each morphogen having a unique characteristic diffusion length. During the equilibration period (DTS < 1500), the parameters $D_{p},\omega_{p},\eta$ and Δt are multiplied by eight in order to speed up the time taken for morphogens to reach a steady state. The faster timescale does not have an effect on morphogenesis because there is no GRN-driven dynamics during the equilibration phase, as described later.

For the simulations presented in the main text, we fixed $N_{morph}=3$ and $N_{TF}=9$. In S1 and S2 and S3 Figs, where we explore variations in network size, the specific values for *N*_*morph*_ and *N*_*TF*_ are explicitly stated.

### Adhesion and membrane tension proteins

Cells encode adhesion proteins constituting *N*_*pairs*_ pairs of lock-and-key proteins that determine cell-cell adhesion and *N*_*med*_ medium proteins that determine cell adhesion to the extracellular medium as well as cell fluidity (explained subsequently). While a simpler homotypic adhesion system, such as that mediated by E-cadherin [72], could have been used, we chose this asymmetric lock-and-key system to provide a more flexible adhesion code to allow a larger array of evolvable interactions between cell states. Each lock protein has a complementary key protein to which it can bind. When two cells are in contact, the adhesion energy between them (*J*_*ij*_ in Equation 1) decreases with the number of expressed pairs of compatible locks and keys (described subsequently). Each pair of lock and key reduces adhesion energy by the same amount. The adhesion energy to the extracellular medium (*J*_*im*_ in Equation 1) decreases with the number of medium proteins expressed by a cell. Medium proteins have graded adhesion strengths (described subsequently). All *J*_*ij*_ and *J*_*im*_ variables used in our model are positive because cell disintegration is favoured when they are negative. When *J*_*ij*_ and/or *J*_*im*_ are small, the energy barriers to cell rearrangements are low and cells behave more fluid-like. When *J*_*ij*_ and/or *J*_*im*_ are high, the energy barriers to cell rearrangements are high and cells behave more solid-like.

Adhesion protein concentrations are Booleanised to an ON or OFF state for adhesion energy calculations (i.e., ON if $x_{p}^{\sigma }>0.5$ else OFF). Specifically, *J*_*ij*_ between neighbouring pixels *i* and *j* that belong to different cells is:


$$\begin{array}{c} J_{ij}=J_{ij}^{\text{max}}-2\sum_{k=1}^{N_{pairs}}\left[\phi_{k}^{ij}+\phi_{k}^{ji}\right] \end{array}$$ (5)

where $\phi_{k}^{ij}=1$ if the *k*-th lock in the cell of pixel *i* and the *k*-th key in the cell of pixel *j* are both ON and otherwise $\phi_{k}^{ij}=0$. Similarly, *J*_*im*_ between pixels *i* and *m* belonging to a cell and the medium, respectively, is:


$$\begin{array}{c} J_{im}=J_{im}^{\text{max}}-\sum_{k=1}^{N_{med}}k\psi_{k} \end{array}$$ (6)

where $\psi_{k}=1$ if the *k*-th medium-adhesion protein is ON, and otherwise $\psi_{k}=0$. $J_{ij}^{\text{max}}=24$ and $J_{im}^{max}=21$ for all simulations. In S16 Fig, we show that the morphogenesis of evolved morphologies is not destroyed by changes to these parameters.

Cells encode *N*_*tens*_ membrane tension proteins that make the cell shape less deformable by energetically constraining it to an elliptic shape. Consequently, cells become more solid-like when they express membrane-tension proteins. Cell shapes are defined as ellipses that have a major axis of length $l_{\sigma }$ constrained to a target length of $L_{\sigma }$ with $\lambda_{L}^{\sigma }$ (see Equation 1). $L_{\sigma }$ increases with the number of expressed membrane tension proteins. The major axis is defined as the longest dimension of the cell, irrespective of its orientation. Because the energetic constraint applies to this dynamically identified major axis without preference for a specific direction, there is no inherent polarity to the cell’s contraction. The orientation and length of the major axis of a cell are re-evaluated after each pixel copy attempt involving that cell. Membrane tension proteins are first Booleanised to an ON or OFF state by the same method used for adhesion proteins. For simulations in the main text, $N_{tens}=2$. When one length protein is ON in cell σ, $L_{\sigma }$ is set to $1.65\sqrt{(}\upsilon_{\sigma })$; when two are ON, $L_{\sigma }$ is set to $1.25\sqrt{(}\upsilon_{\sigma })$ (the numbers 1.65 and 1.25 are arbitrarily chosen). To implement the presence or absence of a length constraint depending on whether membrane tension proteins are expressed, we set $\lambda_{L}^{\sigma }=0.1$ if either membrane tension protein in cell σ is ON, otherwise $\lambda_{L}^{\sigma }=0$.

For the simulations presented in the main text, we fixed $N_{pairs}=5$, $N_{med}=5$ and $N_{tens}=2$. In S1 and S2 and S3 Figs, where we explore variations in network size, the specific values for *N*_*pairs*_, *N*_*med*_ and *N*_*tens*_ are explicitly stated.

We calculated surface tensions for the 24 morphologies that evolved progenitor-cell differentiation in the simulations presented in the main text. These calculations determined the surface tension for three distinct interfaces: (i) between each progenitor-cell type and the medium, (ii) between each differentiated-cell type and the medium, and (iii) between progenitor and differentiated cells. We determined surface tensions, $\gamma_{\tau \tau^{\prime }}$ by the following formula:


$$\begin{array}{c} \gamma_{\tau \tau^{\prime }}=\frac{J_{\tau \tau^{\prime }}}{T}-\frac{J_{\tau \tau }+J_{\tau^{\prime }\tau^{\prime }}}{2T} \end{array}$$ (7)

where $J_{\tau \tau^{\prime }}$ is the adhesion energy arising between cell types τ and $\tau^{\prime }$, while $J_{\tau \tau }$ and $J_{\tau^{\prime }\tau^{\prime }}$ are adhesion energies arising from contact between two cells of the same type, τ and $\tau^{\prime }$, respectively. The parameter *T* is the temperature-like parameter governing the Metropolis algorithm, which scales all energy barriers in the CPM (*T* = 3 for all simulations). For each surface tension calculation, we used the most frequently observed cell state to represent its corresponding cell type. The value of the surface tension between a cell type τ and the medium (*m*) predicts the morphology of cell clusters [34]. If $\gamma_{\tau m}>0$, cell clusters will minimise their surface area and form circular aggregates. If $\gamma_{\tau m}<0$, cells maximise their contact with the medium and disperse from each other. As $\gamma_{\tau m}\to_{\;\;}^{\;\;}0$, the interface with the medium becomes energetically neutral, and the shape of cell clusters is less constrained by adhesion.

### Evolution

To simulate the evolution of morphogenesis, we established an initial population of 60 morphologies with each assigned a different GRN and developing on a separate CPM grid. Each GRN is specified by 234 $Z_{pp^{\prime }}$ values representing the regulatory effects of all transcription factors, as described in Equation 3 (nine transcription factors regulate 26 genes including themselves). The $Z_{pp^{\prime }}$ values of the GRNs assigned to the initial population are randomly generated according to the following probabilities: $P(Z_{pp^{\prime }}=0)=0.54$, $P(Z_{pp^{\prime }}=1)=0.18$, $P(Z_{pp^{\prime }}=-1)=0.18$, $P(Z_{pp^{\prime }}=2)=0.05$ and $P(Z_{pp^{\prime }}=-2)=0.05$. These probability choices are arbitrary. The 15 morphologies with the highest shape complexity reproduce four times to populate the next generation (the definition of shape complexity is described in the next paragraph). Upon reproduction, there is a 50% chance that one of the $Z_{pp^{\prime }}$ values in the GRN mutates. The specific $Z_{pp^{\prime }}$ that mutates is chosen at random with equal probability. The mutation alters the value of $Z_{pp^{\prime }}$, independent of its current value, according to same probabilities used to generate the $Z_{pp^{\prime }}$ assigned to the initial population. Gene duplication and deletion do not occur.

In simulations where morphogen diffusivity mutates (S1 Fig), the values of the diffusion constant, *D*_*p*_ (see Eq. 4) start as a random value taken from a normal distribution between $D_{min}=3\times 10^{-8}$ and $D_{max}=8\times 10^{-7}$. We also change the secretion constant, $\omega_{p}$ (see Eq. 4), to match changes in *D*_*p*_. Specifically, the value of $\omega_{p}$ is equal to:


$$\begin{array}{c} \omega_{p}=\omega_{min}+\epsilon \frac{D_{p}-D_{min}}{D_{max}-D_{min}}, \end{array}$$ (8)

where $\omega_{min}=2.4\times 10^{-3}$ is the minimum value of $\omega_{p}$ and the second term makes $\omega_{p}$ increase with *D*_*p*_, modulated by the constant $\epsilon =1.5\times 10^{-3}$. The purpose of increasing $\omega_{p}$ with *D*_*p*_ is to ensure that the maximum concentration of morphogen $x_{p}^{i}$ in Equation 4 remains close to one. Only *D*_*p*_, not $\omega_{p}$, affects the characteristic length of morphogen diffusion at equilibrium, as described in Materials and methods Section Gene regulatory network and morphogens. Upon reproduction, there is a 25% chance that one of the *D*_*p*_ values mutates, with the choice of *p* occurring randomly with equal probability. The mutation alters the current value of *D*_*p*_ by multiplying it by $e^{-\mu }$, where μ is a random number picked from a normal distribution of mean zero and standard deviation 0.25.

Our measure of shape complexity takes inspiration from an algorithm that quantifies the complexity of two-dimensional shapes [73]. Specifically, we measure shape complexity by summing two measures: the deviation of morphology from a circle (denoted by *z*_1_; S17 Fig) and the degree of inward folds (denoted by *z*_2_; S17 Fig), as described below.

The deviation of a morphology from a circle *z*_1_ is defined by the following equation:


$$\begin{array}{c} z_{1}=\langle |r_{c}-r(\theta )|\rangle_{\theta } \end{array}$$ (9)

where *r*_*c*_ is the hypothetical radius of the morphology if its mass were to be redistributed into a perfect circle, and r(θ) is the maximum distance from the centre of mass of the morphology to any pixel in the direction specified by angle θ (one pixel corresponds to one unit mass). The notation $\langle ...\rangle_{\theta }$ indicates an average taken over all angles θ, where θ is discretised into 360 degrees for computation.

The degree of inward folds (*z*_2_) measures the number and length of concave regions of the morphology. To identify these regions, we begin by drawing horizontal parallel lines across the CPM grid spaced one pixel apart, resulting in a total of 250 lines. Next, we located all segments along the lines that intersect the extracellular medium in between cells. The segments were discarded if they did not exceed a minimum length of 20 pixels to filter out inward folds due to stochastic cell boundary fluctuations. The above procedure was repeated by tilting the 250 parallel lines at 12 evenly-spaced angles across the range [−π/2,π/2]. *z*_2_ is defined as the square root of the total number of the located segments (*z*_2_ is independent of the lengths of the retained segments).

We defined shape complexity as $2z_{1}+2.5z_{2}$. The weights of 2 and 2.5 were selected to ensure that the maximum value of either term is approximately 100 for shapes that are realistically achievable within this model, ensuring that neither term dominates the fitness criterion. In order to filter out noise, shape complexity is taken as an average of 10 evenly-spaced measurements over the last 1,000 DTS. If cells lose physical contact with other cells during development (see S16 Fig for examples), we assign the morphology a fitness of 0 as our quantification of shape complexity is not designed to handle multiple shapes on the same grid. The contributions of deviation of a circle and inward folding to the shape complexity of each morphology is shown in S17 Fig.

We implemented an alternative fitness criterion to determine where progenitor-cell-based morphogenesis is evolutionary accessible (see S1 Appendix and S2 Fig), which has two stages. In the first stage, the fitness criterion is the displacement of a morphology’s centre of mass, measured in pixels, from the start to the end of the 12,000 DTS, denoted *z*_3_. Once the average fitness across all morphologies in a population exceeded 15 pixels, the fitness criterion transitioned to the second stage. In the second stage, the fitness criterion is ($2z_{1}+2.5z_{2})/2+z_{3}^{1.5}$. The weightings of each criterion were selected so that the maximum value of each is approximately 100 for shapes that are realistically achievable within this model, ensuring that neither term dominates the fitness criterion.

To determine whether an evolutionary simulation succeeded or failed, we used fitness thresholds. The thresholds were applied to the morphology that recorded the most complex morphology in the final generation of the simulation. In the simulations selecting only for shape complexity, we applied a threshold of 70 after averaging the shape complexity over 60 developmental replicates in order to account for variance in the complexity score. However, this threshold does not affect our key findings, as there are still highly reproducible morphologies with progenitor-cell differentiation below the threshold (see S5 Fig). In the simulations selecting for both shifting centre of mass and shape complexity, the fitness threshold we used to determine whether an evolutionary simulation succeeded was whether the second stage was reached.

### Cell state and state space

The cell state is an *n*-dimensional Boolean vector, where *n* is the total number of adhesion and membrane tension proteins (although the results do not change when TFs are included in the vector as well). Each element in this vector is the concentration of each protein Booleanised to either ON or OFF (as described previously for lock, key and tension proteins). Each Boolean vector is assigned a single colour on the CPM grid. These colours are chosen arbitrarily, and each morphology has its own distinct colour set. While this approach means that the same cell state might appear in different colours across morphologies, it is uncommon to encounter identical cell states in different evolved morphologies. The cell state of each cell is determined after every numerical integration step of Equation 2. A change in a cell’s Boolean vector corresponds to a cell state transition.

To generate the cell state space, we recorded all cell state transitions for all cells after 6,000 DTS from the beginning of development. Although it makes no qualitative difference when the recording of transitions begins, starting at 6,000 reduces the appearance of “transient” cell states [74], such as the cell state corresponding to the initial conditions of cell proteins, which makes it easier to visualise the cell state space. Cell state transitions are recorded from 10 developmental replicates in order to reduce the effect of noise on cell state space creation. The state space is the directed graph, where the nodes are the cell states and the edges are the transitions. S7 Fig shows two examples of these directed graphs. To simplify the graph, we prune rare transitions (those that occur less than five times across all cells per developmental replicate) and rare cell states. To identify rare cell states, we first count the number of cells in each state after each integration step of Equation 2 to obtain a frequency distribution of cell states. Rare cell states are those that have a frequency of less than 1% from the 10 developmental replicates. However, even without any pruning of cell state transitions and cell states, the cell state space of 22 out of 24 morphologies designated as having progenitor-cell differentiation still exhibit irreversible differentiation (S16 Fig shows one that does not follow this rule).

We used a depth-first search algorithm to identify a graph’s strongly connected components (SCCs). Irreversible differentiation occurs when there is weak connectivity between SCCs (i.e., a path exists from SCC *u* to SCC *v*, but not *v* to *u*). SCCs that do not have incoming paths from any other SCCs (source components) are designated as progenitor-cell types. SCCs that have no outgoing paths to any other SCCs are designated as differentiated-cell types.

### Reproducibility score

We measured the morphogenetic reproducibility of morphologies in a rotation-, reflection- and translation-invariant manner, as follows. We first replayed the development of a morphology 60 times with different random seeds. We then performed pairwise comparisons of all developed morphologies (60×59/2 comparisons). For each pair of morphologies, we computed morphological similarity scores between the two CPM grids on which morphologies developed (denoted by *A* and *B*). We computed the morphological similarity scores (denoted Jac) over many rotations, reflections and translations of grid *B* relative to a fixed grid *A* to find the maximum possible similarity between them (denoted Jac_max_).

To calculate Jac, grids *A* and *B* are transformed from Cartesian to polar coordinates, as follows. The polar coordinate system (r,θ) is discretised into 250 × 360 pixels. Each pixel in (r,θ) is mapped to the pixel closest to (rcosθ,rsinθ) in *A* or *B*, where *r* is the distance from the midpoint of grid *A* or one of 25 equally-spaced locations in grid *B* (thus, multiple pixels in the polar coordinate system can be mapped to the same pixel in Cartesian coordinates). Let $A_{\theta }^{r}$ and $B_{\theta }^{r}$ be one if the mapped pixel at a given (r,θ) coordinate in *A* or *B* belongs to a biological cell and 0 otherwise. We then calculated the Jaccard index, Jac(A,B), which measures the similarity between grids A and B, as follows:


$$\begin{array}{c} \text{Jac}(A,B)=\frac{I_{AB}}{U_{AB}} \end{array}$$ (10)

where $I_{AB}=\sum_{\theta =0^{\circ }}^{359^{\circ }}\sum_{r=0}^{249}r\delta (A_{\theta }^{r},1)\delta (B_{\theta }^{r},1)$ is the number of pixels in the polar coordinate system with a value of one (i.e., the pixel is occupied by a cell) on both A and B, where δ is the Kronecker delta. The multiplication by *r* accounts for the fact that the length of an arc drawn by an increment in θ increases as *r* increases. Similarly, $\begin{array}{c} U_{AB}=\sum_{\theta =0^{\circ }}^{359^{\circ }}\sum_{r=0}^{249}r[\delta (A_{\theta }^{r},1)\delta (B_{\theta }^{r},1)-\delta (A_{\theta }^{r},B_{\theta }^{r})] \end{array}$ is the number of pixels in the polar coordinate system in which $A_{\theta }^{r}=1$ or $B_{\theta }^{r}=1$ (or both). Thus, when $A_{\theta }^{r}$ and $B_{\theta }^{r}$ are exactly the same, $\text{Jac}(A_{\theta }^{r},B_{\theta }^{r})=1$. Next, we shift all values of $B_{\theta }^{r}$ to $B_{\theta +1\;(\mathrm{mod}\;360)}^{r}$, corresponding to a one-degree rotation in Cartesian coordinates, and compute $\text{Jac}(A_{\theta }^{r},B_{\theta }^{r})$ again. We repeat these one-degree rotations 360 times, computing $\text{Jac}(A_{\theta }^{r},B_{\theta }^{r})$ for each. Next, we invert all values of $B_{\theta }^{r}$ to $B_{180-\theta \;(\mathrm{mod}\;360)}^{r}$, which corresponds to a reflection of grid *B*, and repeat the 360 one-degree rotations again, computing $\text{Jac}(A_{\theta }^{r},B_{\theta }^{r})$ for each. Furthermore, we repeated these 720 computations for 25 equally-spaced translations of grid *B* (as mentioned previously), achieved by shifting the location on grid *B* chosen to be *r* = 0 for the polar coordinate system. Translations occur in steps of five pixels at a time to make up a five-by-five square. The maximum $\text{Jac}(A_{\theta }^{r},B_{\theta }^{r})$ recorded across all rotations, reflections and translations for each pairwise comparison is the maximum possible similarity, denoted Jac_max_. The reproducibility score for a morphology is the average Jac_max_ across all 60×59/2 pairwise comparisons.

### Momentum and anisotropy

We defined the momentum, $p_{\sigma }(t)$, of cell σ at time *t* (where *t* is in DTS) as the distance travelled by the cell’s centre of mass between *t* − *t*_*w*_ and *t* mul*t*iplied by the cell’s mass at $t-t_{w}/2$, with unit mass represented by one pixel:


$$\begin{array}{c} p_{\sigma }(t)=m_{\sigma }\left(t-\frac{t_{w}}{2}\right)\frac{s_{\sigma }(t)-s_{\sigma }(t-t_{w})}{t_{w}} \end{array}$$ (11)

where $s_{\sigma }(t)$ is the cell’s centre of mass, $m_{\sigma }(t$) is the cell’s mass at *t*, and *t*_*w*_ is the waiting time. We set *t*_*w*_ to 500 in order to average out *t*he influence of stochastic membrane fluctuations and cell divisions on the centre of mass of the cell, thereby capturing the true mobility of cells. If a cell divides during the waiting time and the σ values of the parent and daughter cells differ, Equation 11 is modified by subtracting the position of the parent cell’s centre of mass ($s_{\sigma }(t-t_{w})$) from the daughter cell’s centre of mass ($s_{\sigma^{\prime }}(t)$), where σ is the index of the parent cell and $\sigma^{\prime }$ is the index of the daughter cell.

In order to separate cell momentum by SCC, we first connected each recording of cell momentum $p_{\sigma }(t)$ to the state of the cell at $t-\frac{t_{w}}{2}$. We then assigned each momentum recording into an SCC depending on that cell state. To create the polar plots of cell momentum magnitude (Fig 4D and 4E), we categorised each momentum measurement assigned to an SCC into one of 36 bins based on its direction. Each bin encompasses momentum measurements within an angular width of 10°. To measure anisotropic motion (Fig 5), we calculated the variance across these 36 bins. To account for total momentum, we divided the variance by the mean momentum across the 36 bins, equal to the dispersion index. The anisotropy value for an SCC is the average of the dispersion indices across five developmental replicates.

## Supporting information

S1 Appendix Text describing the trade-off between reproducibility and shape complexity as well as evolvability of morphogenetic divisions of labour. (PDF)

S1 Movie Morphology-1 development for the 12,000 DTS. (MP4)

S2 Movie Morphology-2 development for the 12,000 DTS. (MP4)

S3 Movie Morphology-6 development for the 12,000 DTS. (MP4)

S4 Movie Morphology-11 development for the 12,000 DTS. (MP4)

S5 Movie Development of a morphology that creates its shape via cell death for the 12,000 DTS. (MP4)

S6 Movie Development of a morphology that branches for the 12,000 DTS. (MP4)

S1 FigEvolution of progenitor-cell differentiation in different kinds of simulations.**(A, B)** Reproducibility and shape complexity of evolved morphologies from simulations where initial condition is a rectangle instead of a circle. Orange triangles are morphologies that evolved progenitor-cell differentiation. Blue diamonds are morphologies that have multiple SCCs but not progenitor-cell differentiation. Black circles are morphologies with a single SCC. Shape complexity is averaged over sixty developmental replicates. The selection pressure for simulations shown in (A) is shape complexity, whereas it is both shape complexity and directional motion in (B), detailed in the Materials and methods. **(C)** Initial shape and cell states of morphologies used for the simulations shown in (A) and (B). The value *D* is the diffusivity of morphogens, which is constant for all simulations at 8 × 10^−7^. **(D, E)** Reproducibility and shape complexity of evolved morphologies from simulations where morphogen diffusivity mutates. Colour and shape coding of data points is the same as (A) and (B). The selection pressure for simulations shown in (C) is shape complexity, whereas it is both shape complexity and directional motion in (D). See Materials and methods for a description of how morphogen diffusivity mutates. **(F)** Initial shape and cell states of morphologies used for the simulations shown in (C) and (D). The morphogen diffusivity mutates over the range $3\times 10^{-8}\leq D\leq 8\times 10^{-6}$. **(G, H)** Two evolved morphologies after 12,000 DTS from simulations shown in (A). (G) has progenitor-cell differentiation; (H) does not. **(I, J)** Two evolved morphologies after 12,000 DTS from simulations shown in (D). (I) has progenitor-cell differentiation; (J) does not. The numbers and types of genes used for all the results presented in this figure were: $N_{morph}=4$, $N_{TF}=10$, $N_{tens}=2$, $N_{pairs}=4$ and $N_{med}=3$.(EPS)

S2 FigReproducible morphogenesis with progenitor-cell differentiation evolves under different selection pressures.**(A)** Reproducibility scores and shape complexity of morphologies evolved under two alternative fitness criteria. Fitness criterion #1 is how much a morphology shifts its centre of mass over the 12,000 DTS (see Materials and methods). Fitness criterion #2 is a sum of the shift in the centre of mass criterion and the shape complexity criterion. Triangles are shape complexity (not fitness) and reproducibility scores of evolved morphologies from 25 simulations using fitness criterion #1. Circles are shape complexity and reproducibility scores of evolved morphologies from 31 simulations using fitness criterion #2. Data colour-coded orange indicates morphologies with progenitor-cell differentiation. Data colour-coded black indicates morphologies without progenitor-cell differentiation. The dashed line is a shape complexity of 70, the threshold we used to determine if a morphology was sufficiently morphologically complex in the original set of simulations, where the selection pressure was only shape complexity (see Materials and methods). The dashed line at a reproducibility score of 66% is the cut-off for high reproducibility indicated in Fig 2A in the main text. **(B)** Three evolved morphologies after 12,000 DTS from simulations using fitness criterion #1 that did not evolve progenitor-cell differentiation. **(C)** Three evolved morphologies after 12,000 DTS from simulations using fitness criterion #1 that did evolve progenitor-cell differentiation. **(D)** Three evolved morphologies after 12,000 DTS from simulations using fitness criterion #2 that did evolve progenitor-cell differentiation. **(E)** One of the two evolved morphologies without progenitor-cell differentiation from the simulations using fitness criterion #2, shown after 12,000 DTS. Its state space is shown to the right. Although the state space shows multiple SCCs, there is no unidirectional transition between them and thus no progenitor-cell differentiation. The morphology is poorly reproducible because one of its SCCs has both moving and stationary states in it. **(F, G)** Development of two morphologies with progenitor-cell differentiation. Morphologies are shown after 2,000, 6,000 and 12,000 DTS. Simplified cell state spaces are shown to the right, with these cell state spaces indicating multiple SCCs with unidirectional transitions. **(H)** The rate at which cells divide per developmental time when their state belongs to an upstream SCC (left) or a downstream SCC (right). Each data point represents an SCC from the 29 evolved morphologies that had multiple SCCs with unidirectional transitions that were evolved under selection for directional motion and shape complexity. Black lines connect upstream SCCs to their counterpart downstream SCCs. Boxes show medians and interquartile ranges. The number and types of genes for simulations presented in this figure is the same as the main text ($N_{morph}=3$, $N_{TF}=9$, $N_{tens}=2$, $N_{pairs}=5$ and $N_{med}=5$.).(EPS)

S3 FigEvolution of progenitor-cell differentiation and high reproducibility with a minimal genome.**(A)** Reproducibility scores and shape complexity of morphologies evolved under two alternative fitness criteria using a minimal genome model. Fitness criterion #1 is how much a morphology shifts its centre of mass over the 12,000 DTS (see Materials and methods). Fitness criterion #2 is a sum of the shift in the centre of mass criterion and the shape complexity criterion (see Materials and methods). Triangles are shape complexity (not fitness) and reproducibility scores of evolved morphologies from eight simulations using fitness criterion #1. Circles are shape complexity and reproducibility scores of evolved morphologies from seven simulations using fitness criterion #2. Data colour-coded orange indicates morphologies with progenitor-cell differentiation. Data colour-coded black indicates morphologies without progenitor-cell differentiation. The dashed line is a shape complexity of 70, the threshold we used to determine if a morphology was sufficiently morphologically complex in the original set of simulations, where the selection pressure was only shape complexity (see Materials and methods). The dashed line at a reproducibility score of 66% is the cut-off for high reproducibility indicated in Fig 2A in the main text. The minimal genome comprises 12 genes (as opposed to 26): $N_{morph}=3$, $N_{TF}=6$, $N_{tens}=0$, $N_{pairs}=2$ and $N_{med}=2$. The adhesion parameters that we changed to accommodate the smaller genome are $J_{ij}^{max}=20$, $\phi_{k}^{ij}=4$, $J_{im}^{max}=18$, $\psi_{1}=10$ and $\psi_{2}=2$. There are no membrane tension proteins. **(B)** A highly reproducible morphology developed for 12,000 DTS from the minimal genome model with progenitor-cell differentiation. Its state space to the right shows multiple SCCs connected by a unidirectional transition. **(C)** A poorly reproducible morphology developed for 12,000 DTS from the minimal genome model without progenitor-cell differentiation. Its state space to the right shows a single SCC.(EPS)

S4 Fig Evolution and evolved morphologies. **A-C** Three plots of fitness evolution, each from a separate simulation where morphologies with high reproducibility evolved. The black lines show the maximum recorded fitness at each generation. The orange lines show the average fitness of each generation. Above each plot are six or seven morphologies at the end of their development (12,000 DTS). Each morphology is taken from a separate generation and is the most morphologically complex in its generation, shown approximately above the generation it belongs to. **(D, E)** Two plots of fitness evolution, each from a separate simulation where morphologies with poor reproducibility evolved, mirroring the plots shown in (A-C). The evolutionary trajectories shown in (A-E) indicate that fitness reaches a plateau before 2.5 × 10^3^ generations in 5 out of 6 simulations (we ran all simulations for at least this number of generations). Plateaus appear to persist for a long time, as exemplified by (C), which we ran for 11,500 generations. We did not run simulations for >10,000 generations due to computational limitations (10,000 generations takes approximately one week with 60 CPUs). The similarity of the morphologies above each plot indicates that the phenotype becomes conserved through evolution. This fixation implies that the evolved morphologies accurately represent the evolutionary outcome of a simulation. **(F)** Morphological variation in a population due to mutation. Each morphology is from the final generation from the simulation shown in (C). Each morphology has a unique GRN topology due to mutations. **(G)** Zoo of highly reproducible and **(H)** poorly reproducible morphologies, each shown at the end of their development (12,000 DTS). Each morphology is a different evolved morphology not shown in the main text.
(EPS)

S5 Fig Evolved morphologies that did not reach the complexity threshold. **(A)** Reproducibility scores of 16 of the 36 evolved morphologies that did not reach the threshold for shape complexity. We chose these 16 because their shape complexity is above a score of 40 but below 70 (70 is the threshold we used to determine if a morphology has a complex morphology in the original 126 simulations). The remaining 19 morphologies that have scores below 40 were excluded because they have circular shapes. Black circles are morphologies that have state spaces with a single SCC, of which there are seven. The morphology indicated by the blue diamond has a state space with multiple SCCs with no unidirectional transitions. It has a similar morphogenesis to the morphology shown in S6 Fig. Orange triangles are morphologies with progenitor-cell differentiation, of which there are eight. Categories of “high”, “intermediate” and “poor” are copied from Fig 2A in the main text. The eight morphologies with progenitor-cell differentiation were significantly more reproducible than those without (*p* = 0.007, two-sample t-test). The arrows point to the morphologies shown in (B) and (C). **(B, C)** Development of evolved morphologies that did reach the complexity threshold shown at four successive time points.
(EPS)

S6 Fig Diversity of developmental dynamics in our evolved model. **(A, B)** Development of two morphologies categorised as having both a complex and reproducible morphology that do not have progenitor-cell differentiation, each shown at four successive time points during their development. Their states spaces show cell states partitioned into two strongly connected components (SCCs, grey boxes) with no unidirectional transitions. The morphology undergoes morphogenesis by stacking “balls” of cells on top of each other, with differential adhesion between cells from different balls keeping each ball separated. Both morphologies are highly reproducible because all cells are classed as moving, i.e., there are no stationary cells. The morphology in (A) is indicated by a blue diamond in Fig 2A. The morphology in (B) is indicated by a blue diamond in S1 Fig. **(C, D)** Development of two morphologies with progenitor-cell differentiation that undergo branching, each shown at four successive time points during their development. We categorise this type of morphogenesis as branching instead of a bifurcation because it occurs consistently over developmental replicates. Branching occurs by progenitor-cell differentiation at the tip, which splits the group of progenitor cells in two. **(E)** Development of a morphology with progenitor-cell differentiation that undergoes epiboly shown at four DTS during its development. The orange progenitor cells spread out over the surface of the morphology before differentiating at the top and bottom edge. The morphology is highly reproducible (S1 Fig) **(F)** Development of a morphology where cells die to produce protrusions, shown at four successive time points during its development. Its development is shown alongside a state space that shows it has only a single SCC. **(G)** Development of the same morphology shown in (F) except with three times as many initial cells, shown at five successive time points during its development (with development extended to 28,000 DTS). The development shows that the protrusions have a characteristic width of 2–3 cells wide. **(H)** Schematic indicating the development dynamics of the morphology shown in (FG). When the width of the morphology is larger than the characteristic width, cells in the yellow state ingress into the centre where they transition to orange cells. The orange cells are squished by the grey cells and die. The blue cells proliferate to extend the protrusions. The morphology is poorly reproducible (see S1 Fig).
(EPS)

S7 Fig Highly and poorly reproducible morphologies have different cell-state transition dynamics. Panels A through D correspond to morphology-1 (poorly reproducible), and panels E through H to morphology-2 (highly reproducible). **(A, E)** Morphologies of morphology-1 and morphology-2 after the 12,000 DTS. **(B, F)** Above the graphs are coloured circles depicting cell-state transitions for one cell, indicated by the asterisks in panels A and E, over the 12,000 DTS (some cell-state transitions are excluded for visibility). The top graphs show the concentrations of eight cell adhesion proteins over the 12,000 DTS. The bottom graphs show cell size over the 12,000 DTS. Daggers (†) indicate drops in size caused by cell division. **(C, G)** Simplified cell state spaces consisting of cell states (nodes) and cell-state transitions (arrows). Node sizes depict cell state frequency. Cell states are partitioned into SCCs (coloured boxes). **(D, H)** Visualisations of cell states (colours) mapped onto cell protein expression profiles (data points) that have undergone dimension reduction by UMAP. The data consists of 85,825 (D) and 115,625 (H) points, each collected from every cell at intervals of 40 DTS. **(I)** The state space shown in (C) without any pruning of cell states and cell state transitions. Many SCCs in the unpruned cell state space are “transitory”. These transitory SCCs consist of cell states observed during differentiation from SCC-1 to SCC-2. **(J)** The state space shown in (G) without pruning of cell states and cell state transitions. **(K)** Morphologies 3, 4 and 5 from the main text are shown at the end of their respective developments (12,000 DTS), along with their simplified state spaces. The layout of the cell states in the morphology mirrors the layout of the cell states in the state space. The green box in the morphology-5 state space corresponds to an SCC disconnected from the other two (i.e., no unidirectional transitions).
(EPS)

S8 Fig Morphogenesis of a morphology with intermediate reproducibility. **(A, B)** Developmental replicates at three time points. The vector plots show the displacement of the centre of mass of each cell over the previous 2,000 DTS at each respective time point, with colours indicating magnitude (the lighter, the larger). **(C)** Simplified state space of this intermediately reproducible morphology. The state space shows one progenitor-cell type (SCC-1), one differentiated-cell type (SCC-2) and one SCC (SCC-3) that contains neither progenitor nor differentiated cells. The regions of the morphologies corresponding to the progenitor and differentiated cell types appear morphologically similar between developmental replicates. In contrast, the region corresponding to cells in SCC-3 appears dissimilar between developmental replicates. This dissimilarity arises because there is a bifurcation in the motion of these cells in replicate 2 but not in replicate 1 (dashed black arrows). This bifurcation occurs because both moving and stationary states are both contained in SCC-3, as the vector plots confirm. Thus, this morphology’s development shows properties of both highly (SCC-1 and SCC-2) and poorly (SCC-3) reproducible morphogenesis.
(EPS)

S9 Fig Components of shape complexity across evolved morphologies. We measured shape complexity as a sum of two components: deviation from a circle and inward folding (see Materials and methods in the main text). **(A)** Contributions from deviation from a circle (left) and inward folding (right) to shape complexity across evolved morphologies. Evolved morphologies are separated into those with progenitor-cell differentiation (orange triangles) and those without (black circles). Boxes show medians and interquartile ranges (IQR) for each grouping. Each data point is an average over 60 developmental replicates. **(B)** Coefficient of variation (normalised standard deviation) in the contributions from deviation from a circle (left) and inward folding (right) to shape complexity across all evolved morphologies. Groupings and boxes are the same as in (A). Coefficient of variation is defined as the standard deviation divided by the average over the 60 developmental replicates. A Mann-Whitney U-test comparing whether morphologies with progenitor-cell differentiation vs morphologies without progenitor-cell differentiation have the same distribution in (B) gives *p* = 0.04 for deviation from a circle and *p* = 0.002 for inward folding. These p-values indicate that morphologies with progenitor-cell differentiation tend to have lower variability in their shape complexity compared to those without.
(EPS)

S10 Fig The trade-off between shape complexity and reproducibility. **(A)** Reproducibility scores of the 65 morphologies without progenitor-cell differentiation (black circles) and 24 evolved morphologies with progenitor-cell differentiation (PCD, orange triangles). The unfilled box plot shows the median and interquartile range of reproducibility scores for each grouping. **(B)** Group similarity scores of the 65 evolved morphologies without progenitor-cell differentiation (left green box plot, *n* = 2211) and the 24 with progenitor-cell differentiation (right green box plot, *n* = 276). Boxes show medians and interquartile ranges (IQR). Whiskers show the range. **(C)** Scatter plot of reproducibility scores against shape complexity for the 90 evolved morphologies. Data points are coloured and shaped as in (D). The black and orange dashed lines are the regression for poorly reproducible and highly reproducible morphologies described in S1 Appendix. **(D)** Scatter plot of reproducibility scores against shape complexity scores throughout evolutionary simulations. Each data point is the most complex morphologies in a population from a generation, taken at intervals of 100 generations. The orange data points are morphologies from simulations where highly reproducible morphologies with progenitor-cell differentiation evolved (18 simulations, 1508 data points). Black data points are from simulations where poorly reproducible morphologies evolved (18 simulations, 1180 data points). Each line is the linear regression performed on all data points from the same simulation, coloured in the same way as the data points. Performing a single linear regression across all orange data points gives a 95% confidence interval on the slope of −0.0011 to −0.0099 (*R*^2^ = 0.32) and across all black points gives −0.0032 to −0.0031 (*R*^2^ = 0.86). **(D)** Evolution of reproducibility (dotted lines) and fitness (solid lines) in a simulation where progenitor-cell differentiation evolved (orange) and a simulation where one did not (black). The data used to generate the lines is the shape complexity and reproducibility of the highest-fitness morphology in populations at intervals of 100 generations for each simulation. Progenitor-cell differentiation is first observed in the simulation in orange at generation 500.
(EPS)

S11 Fig Number of developmental cell states does not predict shape complexity or morphogenetic reproducibility. **(A)** Shape complexity of each evolved morphology as a function of number of cell states. **(B)** Reproducibility score of each evolved morphology as a function of number of cell states. A cell state is counted towards the total if it is observed at any point in the development of a morphology over 10 developmental replicates, excluding the four possible initial states. Orange triangles are those classified as having high or intermediate reproducibility in the main text (Fig 2A). Black circles are those classified as having poor reproducibility in the main text (Fig 2A). For morphologies classified as highly reproducible, a regression of shape complexity against number of cell states gives *R*^2^ = 0.02, with a non-significant p-value on the slope (*p* = 0.56). A regression of reproducibility against number of cell states gives *R*^2^ = 0.16, with a non-significant p-value on the slope (*p* = 0.07). For those classified as poorly reproducible morphology, a regression of shape complexity against number of cell states gives *R*^2^ = 0.04, with a non-significant p-value on the slope (*p* = 0.12). A regression of reproducibility against number of cell states gives *R*^2^ = 0.00, with a non-significant p-value on the slope (*p* = 0.84).
(EPS)

S12 Fig The causes of poor morphogenetic reproducibility. **(A)** Developmental replicate of morphology-1 from the main text depicted at six successive time points. Vector plots show the displacement of the centre of mass of each cell during 2,000 DTS at each respective time point, with colours indicating magnitude (the lighter, the larger). **(B)** A perturbed development of morphology-1 using the same random seed as (A) to show that moving-to-stationary transitions cause bifurcations. Development was perturbed by artificially changing the protein expression profiles of three moving cells at the bottom of the morphology to those of a stationary cell state (blue cells next to the asterisk). The protein expression profiles were taken from an arbitrarily chosen blue cell. We also changed the morphogen concentrations overlaying the three cells to that of the arbitrarily chosen blue cell. We performed this perturbation after 4,100 DTS. This perturbation resulted in a bifurcation of collective cell motion, as indicated by the vector plots and the morphology. **(C)** A perturbed development of morphology-1 using the same random seed as (A) to show that stationary-to-moving transitions cause protrusions. Development was perturbed by artificially changing the protein expression profiles of three stationary cells at the left flank of the morphology to those of a moving cell state (grey cells next to the asterisk). The protein expression profiles were taken from an arbitrarily chosen grey cell. We also changed the morphogen concentrations overlaying the three cells to that of the arbitrarily chosen grey cell. We performed this perturbation after 4,100 DTS. This perturbation resulted in a protrusion, as indicated by the vector plots.
(EPS)

S13 FigAdhesive properties of morphologies with progenitor-cell differentiation.**(A)** Adhesion energy arising from progenitor-to-progenitor cell contacts, progenitor-to-differentiated cell contacts and differentiated-to-differentiated cell contacts across the 30 kinds of progenitor-cell differentiation from the 24 morphologies that display it. The grey lines connect adhesion energies between a progenitor cell type and the differentiated cell type it transitions to. Thicker grey lines indicate data overlap. Adhesion energies are the *J*_*ij*_ values arising from contact between the most frequently observed state of each cell type to itself (progenitor-to-progenitor or differentiated-to-differentiated) or the most frequently observed progenitor-cell state to its corresponding most frequently observed differentiated-cell state (progenitor-to-differentiated). Boxes show medians and interquartile ranges across the 30 kinds of progenitor-cell differentiation (the 25th and 75th percentiles are equal to the median for progenitor-to-progenitor and differentiated-to-differentiated adhesion energies; the 75th percentile is equal to the median for differentiated-to-differentiated adhesion energies). The *y*-axis plots the adhesion energies divided by the temperature-like parameter *T* (set to *T* = 3 for all simulations), since this parameter rescales all energy barriers in the CPM. **(B)** Progenitor-to-medium surface tension (*n* = 30), progenitor-to-differentiated surface tension (*n* = 30) and differentiated-to-medium surface tension (*n* = 26) across the 24 morphologies that evolved progenitor-cell differentiation in main text simulations. The median equals the 25th percentile (bottom of the box) because most progenitor-cell types have γ=1.33, and most differentiated-cell types have γ=0. See Materials and methods in the main text for an explanation of surface tensions.(EPS)

S14 Fig Progenitor-cell motion and divisions are isotropic when isolated from other cell types. **(A)** Four evolved morphologies with progenitor-cell differentiation, shown at their developmental endpoints (12,000 DTS). Each morphology has one progenitor-cell type and one differentiated-cell type. **(B, C)** Polar plots of momentum magnitude by angle of momentum over normal development of the four morphologies, separated by cell type; (B) shows plots for the four progenitor-cell types, where momentum appears anisotropic, and (C) shows plots for the four differentiated-cell types. **(D)** Isolated progenitor cells for each morphology at their developmental endpoints (12,000 DTS). **(E)** Polar plots of momentum magnitude by the angle of momentum of the isolated progenitor cells for each morphology, showing radially symmetrically distributed motion. **(F)** Isolated differentiated cells for each morphology at their developmental endpoints (12,000 DTS). **(G)** Polar plots of momentum magnitude by angle of motion of the isolated differentiated cells for each morphology. **(H, I, J)** Analysis of progenitor-cell differentiation in morphology-1. We show morphology-1 here because its progenitor-cell type is one of the four (out of 30) that differentiates when isolated from other cell types. (H) shows morphogen concentrations along a cross-section of morphology-1 at 8,000 DTS. The sum of cell protein concentrations is also shown for cells along this cross-section (each cross is one cell). The green morphogen induces progenitor-cell differentiation, which is produced by differentiated cells. However, progenitor cells also begin to express the green morphogen themselves if the black and red morphogen concentrations get too high. (I) shows the development of isolated type-1 progenitor cells after 2000, 4000, 6000 and 12,000 DTS. The isolated progenitor cells differentiate around the cluster’s centre around 4,000 DTS. Differentiation begins at the cluster’s centre because black and red morphogens are at their highest concentration, resulting in the expression of the green morphogen that induces differentiation. The differentiation of progenitor cells in this isolated morphology causes progenitor and differentiated cells to split apart due to differential adhesion between progenitor and differentiated cells. **(J)** Development of morphology-1 progenitor cells in isolation, except with morphogen concentrations prevented from increasing above 1.0. Preventing black and red morphogen concentrations from increasing above 1.0 stops the green morphogen from being expressed and thus prevents progenitor-cell differentiation. For the four progenitor-cell types that autonomously differentiate, including this one, we measured the motion anisotropy of isolated progenitor cells (plotted in Fig 5) after placing restrictions on morphogen concentrations to prevent differentiation. The asterisk in (D, E) shows the morphology (D) and polar plot (E) of isolated progenitor-cell motion from morphology-1 when these morphogen restrictions are in place. **(K, L)** Spatial distribution of cell divisions (black dots) and differentiations (orange dots) for (C) type-1 and (D) type-2 progenitor-cell domains from morphology-6, with the pole of the plots defined as the centre of mass of all of the respective progenitor cells in that cluster (i.e., the pole shifts as progenitor cells move over development). The location of a cell division is marked at the centre of mass of the parent cell. The plots show that type-1 progenitor cell divisions are asymmetric in that they occur around the cluster’s periphery rather than the centre. In contrast, type-2 progenitor cell divisions predominantly occur distal to the location of progenitor-cell differentiation. **(M, N)** Spatial distribution of cell divisions for type-1 and type-2 progenitor cells from morphology-6, as in (K, L).
(EPS)

S15 Fig Programmability of progenitor-cell-based morphogenesis. We created arbitrary morphologies of progenitor and differentiated cells from morphology-1 and morphology-6 and simulated their development for morphology-1 and morphology-6. **(A)** Development of an arbitrary morphology that begins as a triangular shape of differentiated cells from morphology-6 accompanied by a small domain of type-2 progenitor cells from that morphology on the diagonal surface, with development extended to 15,000 DTS. We programmed this morphology by artificially changing the state of the initial cells to either differentiated cells or progenitor cells depending on their initial position in the triangle. **(B)** The same developmental trajectory of morphology-6 as shown in Fig 4A of the main text, except with the state of the differentiated cells on the centre-right flank artificially changed to type-2 progenitor cells at 7,000 DTS. This artificial state-change causes a new branch to form perpendicular to the native one. **(C)** An arbitrary morphology that begins as a triangular shape of differentiated cells from morphology-6 accompanied by a small domain of progenitor cells from that morphology on the diagonal surface, with development lasting for 10,000 DTS.
(EPS)

S16 Fig Technical details of the model and analysis. **(A, B, C, D)** Robustness of morphology-6 morphogenesis to changes in temperature and adhesion parameters. (A) shows the normal development of morphology-6 after 2000, 6000 and 12,000 DTS. (B) shows the development of morphology-6 using the same random seed as (A) but with the temperature, *T*, decreased by 0.5 (where *T* = 3 by default). (C) shows the same development of morphology-6 using the same random seed as (A) but with *T* increased by 0.5. (D) shows the same development of morphology-6 using the same random seed as (A) but with all cell-cell adhesion energies, *J*_*ij*_, decreased by one. **(E, F)** Morphologies that are designated 0 fitness because there is no single shape to measure the complexity of. We show the development of two morphologies at four successive time points. The cells of both morphologies separate from each other during development. These morphologies were arbitrarily chosen from evolving populations in evolutionary simulations. **(G, H, I, J)** One of the two morphologies with progenitor-cell differentiation that has rare transitions from differentiated to progenitor cells (these rare transitions were removed during pruning). (G) shows a developmental replicate of the morphology, and (H) shows its state space after pruning, indicating multiple SCCs with unidirectional transitions. (I) shows the same state space before the pruning of rare transitions (although rare cell states are pruned for clarity), indicating only a single SCC. (J) is a visualisation of cell states (colours) mapped onto cell protein expression profiles (points) that have undergone dimension reduction by UMAP for the morphology shown in (G). Profiles are collected from four developmental replicates. Two lines in the UMAP plot connect the white cell state (deemed to be differentiated in the pruned state space) to other cell states. The thinner of the two lines corresponds to a transition of this differentiated cell state to the other differentiated cell state. This transition is one of those removed after pruning.
(EPS)

S17 Fig Visual illustration of algorithm used to quantify shape complexity. **(A)** Visualization of the measure of deviation from a circle. We show the difference between the actual morphology radius (*r*_1_) and the radius if all morphology pixels were to be circularly distributed (*r*_2_, black circle) at two locations. **(B)** Visualization of the measure of inward folds. Solid black lines depict the evenly spaced parallel lines drawn across the grid at one of the 12 evenly-spaced angles. The actual density of lines occur is much higher density than the five shown here. The orange segments of the lines indicate “gaps” in the morphology. The square root of the total number of these gaps with a minimum length of 20 pixels is taken as the degree of inward folding.
(EPS)

## References

[1] Hogan BL. Morphogenesis. Cell. 1999;96(2):225–33. 9988217 10.1016/s0092-8674(00)80562-0

[2] Wolpert L, Tickle C, Arias AM. Principles of Development. USA: Oxford University Press; 2015.

[3] Goodwin BC, Kauffman S, Murray JD. Is morphogenesis an intrinsically robust process? J Theor Biol. 1993;163(1):135–44. doi: 10.1006/jtbi.1993.1112 8412239

[4] Kauffman SA, et al. The origins of order: Self-organization and selection in evolution. USA: Oxford University Press; 1993.

[5] Tautz D. Segmentation. Dev Cell. 2004;7(3):301–12. 15363406 10.1016/j.devcel.2004.08.008

[6] Staps M, Miller PW, Tarnita CE, Mallarino R. Development shapes the evolutionary diversification of rodent stripe patterns. Proc Natl Acad Sci U S A. 2023;120(45):e2312077120. doi: 10.1073/pnas.2312077120 37871159 PMC10636316

[7] Zuniga A. Next generation limb development and evolution: old questions, new perspectives. Development. 2015;142(22):3810–20. doi: 10.1242/dev.125757 26577204

[8] Iruela-Arispe ML, Beitel GJ. Tubulogenesis. Development. 2013;140(14):2851–5. 23821032 10.1242/dev.070680 PMC3699276

[9] Miao Y, Djeffal Y, De Simone A, Zhu K, Lee JG, Lu Z, et al. Reconstruction and deconstruction of human somitogenesis in vitro. Nature. 2023;614(7948):500–8. doi: 10.1038/s41586-022-05655-4 36543321 PMC10018515

[10] Aristotle. The complete works of Aristotle—The revised Oxford translation. Princeton University Press; 1984.

[11] Félix M-A, Barkoulas M. Pervasive robustness in biological systems. Nat Rev Genet. 2015;16(8):483–96. doi: 10.1038/nrg3949 26184598

[12] Green RM, Fish JL, Young NM, Smith FJ, Roberts B, Dolan K, et al. Developmental nonlinearity drives phenotypic robustness. Nat Commun. 2017;8(1):1970. doi: 10.1038/s41467-017-02037-7 29213092 PMC5719035

[13] Osterwalder M, Barozzi I, Tissières V, Fukuda-Yuzawa Y, Mannion BJ, Afzal SY, et al. Enhancer redundancy provides phenotypic robustness in mammalian development. Nature. 2018;554(7691):239–43. doi: 10.1038/nature25461 29420474 PMC5808607

[14] Swain PS, Elowitz MB, Siggia ED. Intrinsic and extrinsic contributions to stochasticity in gene expression. Proc Natl Acad Sci U S A. 2002;99(20):12795–800. doi: 10.1073/pnas.162041399 12237400 PMC130539

[15] Tsimring LS. Noise in biology. Rep Prog Phys. 2014;77(2):026601. doi: 10.1088/0034-4885/77/2/026601 24444693 PMC4033672

[16] Briscoe J, Small S. Morphogen rules: design principles of gradient-mediated embryo patterning. Development. 2015;142(23):3996–4009. doi: 10.1242/dev.129452 26628090 PMC4712844

[17] Crampin EJ, Gaffney EA, Maini PK. Reaction and diffusion on growing domains: scenarios for robust pattern formation. Bull Math Biol. 1999;61(6):1093–120. doi: 10.1006/bulm.1999.0131 17879872

[18] Freeman M. Feedback control of intercellular signalling in development. Nature. 2000;408(6810):313–9. doi: 10.1038/35042500 11099031

[19] Eldar A, Dorfman R, Weiss D, Ashe H, Shilo B-Z, Barkai N. Robustness of the BMP morphogen gradient in Drosophila embryonic patterning. Nature. 2002;419(6904):304–8. doi: 10.1038/nature01061 12239569

[20] Kitano H. Biological robustness. Nat Rev Genet. 2004;5(11):826–37. doi: 10.1038/nrg1471 15520792

[21] Rogers KW, Schier AF. Morphogen gradients: from generation to interpretation. Annu Rev Cell Dev Biol. 2011;27:377–407. doi: 10.1146/annurev-cellbio-092910-154148 21801015

[22] Madamanchi A, Mullins MC, Umulis DM. Diversity and robustness of bone morphogenetic protein pattern formation. Development. 2021;148(7):dev192344. doi: 10.1242/dev.192344 33795238 PMC8034876

[23] Reinitz J, Vakulenko S, Sudakow I, Grigoriev D. Robust morphogenesis by chaotic dynamics. Sci Rep. 2023;13(1):7482. doi: 10.1038/s41598-023-34041-x 37160971 PMC10170119

[24] Majka M, Becker NB, Ten Wolde PR, Zagorski M, Sokolowski TR. Stable developmental patterns of gene expression without morphogen gradients. PLoS Comput Biol. 2024;20(12):e1012555. doi: 10.1371/journal.pcbi.1012555 39621779 PMC11661646

[25] Gilmour D, Rembold M, Leptin M. From morphogen to morphogenesis and back. Nature. 2017;541(7637):311–20. doi: 10.1038/nature21348 28102269

[26] Hagolani PF, Zimm R, Marin-Riera M, Salazar-Ciudad I. Cell signaling stabilizes morphogenesis against noise. Development. 2019;146(20):dev179309. doi: 10.1242/dev.179309 31628213

[27] Cano-Fernández H, Tissot T, Brun-Usan M, Salazar-Ciudad I. On the origins of developmental robustness: modeling buffering mechanisms against cell-level noise. Development. 2023;150(24):dev201911. doi: 10.1242/dev.201911 38032004

[28] Hogeweg P. On searching generic properties of non-generic phenomena: an approach to bioinformatic theory formation. Artificial life VI. Cambridge, MA: MIT Press; 1998. p. 285–94.

[29] Hogeweg P. Evolving mechanisms of morphogenesis: on the interplay between differential adhesion and cell differentiation. J Theor Biol. 2000;203(4):317–33. doi: 10.1006/jtbi.2000.1087 10736211

[30] Hogeweg P. Shapes in the shadow: evolutionary dynamics of morphogenesis. Artif Life. 2000;6(1):85–101. doi: 10.1162/106454600568339 10943667

[31] Ten Tusscher KH, Hogeweg P. Evolution of networks for body plan patterning; interplay of modularity, robustness and evolvability. PLoS Comput Biol. 2011;7(10):e1002208. doi: 10.1371/journal.pcbi.1002208 21998573 PMC3188509

[32] Vroomans RM, Hogeweg P, Ten Tusscher KH. In silico evo-devo: reconstructing stages in the evolution of animal segmentation. EvoDevo. 2016;7:1–20. 27482374 10.1186/s13227-016-0052-8 PMC4968448

[33] Vroomans RMA, Hogeweg P, Ten Tusscher KHWJ. Around the clock: gradient shape and noise impact the evolution of oscillatory segmentation dynamics. Evodevo. 2018;9:24. doi: 10.1186/s13227-018-0113-2 30555670 PMC6288972

[34] Graner F, Glazier J. Simulation of biological cell sorting using a two-dimensional extended Potts model. Phys Rev Lett. 1992;69(13):2013–6. doi: 10.1103/PhysRevLett.69.2013 10046374

[35] Hirashima T, Rens EG, Merks RMH. Cellular Potts modeling of complex multicellular behaviors in tissue morphogenesis. Dev Growth Differ. 2017;59(5):329–39. doi: 10.1111/dgd.12358 28593653

[36] Rozario T, DeSimone DW. The extracellular matrix in development and morphogenesis: a dynamic view. Dev Biol. 2010;341(1):126–40. doi: 10.1016/j.ydbio.2009.10.026 19854168 PMC2854274

[37] Chen CS, Mrksich M, Huang S, Whitesides GM, Ingber DE. Geometric control of cell life and death. Science. 1997;276(5317):1425–8. doi: 10.1126/science.276.5317.1425 9162012

[38] Nelson CM, Jean RP, Tan JL, Liu WF, Sniadecki NJ, Spector AA, et al. Emergent patterns of growth controlled by multicellular form and mechanics. Proc Natl Acad Sci U S A. 2005;102(33):11594–9. doi: 10.1073/pnas.0502575102 16049098 PMC1187971

[39] Guillot C, Lecuit T. Mechanics of epithelial tissue homeostasis and morphogenesis. Science. 2013;340(6137):1185–9. doi: 10.1126/science.1235249 23744939

[40] Chiang M, Marenduzzo D. Glass transitions in the cellular Potts model. EPL. 2016;116(2):28009. doi: 10.1209/0295-5075/116/28009

[41] Steinberg MS. Differential adhesion in morphogenesis: a modern view. Curr Opin Genet Dev. 2007;17(4):281–6. doi: 10.1016/j.gde.2007.05.002 17624758

[42] Sehring IM, Dong B, Denker E, Bhattachan P, Deng W, Mathiesen BT, et al. An equatorial contractile mechanism drives cell elongation but not cell division. PLoS Biol. 2014;12(2):e1001781. doi: 10.1371/journal.pbio.1001781 24503569 PMC3913557

[43] Knabe JF, Schilstra MJ, Nehaniv CL. Evolution and morphogenesis of differentiated multicellular organisms: autonomously generated diffusion gradients for positional information. In: Artificial Life XI, 2008.

[44] Colizzi ES, Vroomans RM, Merks RM. Evolution of multicellularity by collective integration of spatial information. Elife. 2020;9:e56349. doi: 10.7554/eLife.56349 33064078 PMC7652420

[45] Hirway SU, Lemmon CA, Weinberg SH. Multicellular mechanochemical hybrid cellular Potts model of tissue formation during epithelial‐mesenchymal transition. Comp Sys Onco. 2021;1(4):e1031. doi: 10.1002/cso2.1031

[46] Vroomans RMA, Colizzi ES. Evolution of selfish multicellularity: collective organisation of individual spatio-temporal regulatory strategies. BMC Ecol Evol. 2023;23(1):35. doi: 10.1186/s12862-023-02133-x 37468829 PMC10357660

[47] Martin BL, Kimelman D. Canonical Wnt signaling dynamically controls multiple stem cell fate decisions during vertebrate body formation. Dev Cell. 2012;22(1):223–32. doi: 10.1016/j.devcel.2011.11.001 22264734 PMC3465166

[48] Farin HF, Jordens I, Mosa MH, Basak O, Korving J, Tauriello DVF, et al. Visualization of a short-range Wnt gradient in the intestinal stem-cell niche. Nature. 2016;530(7590):340–3. doi: 10.1038/nature16937 26863187

[49] Zhang HT, Hiiragi T. Symmetry Breaking in the Mammalian Embryo. Annu Rev Cell Dev Biol. 2018;34:405–26. doi: 10.1146/annurev-cellbio-100617-062616 30095292

[50] Bruce AEE. Zebrafish epiboly: Spreading thin over the yolk. Dev Dyn. 2016;245(3):244–58. doi: 10.1002/dvdy.24353 26434660

[51] Mizuno K, Hirashima T, Toda S. Robust tissue pattern formation by coupling morphogen signal and cell adhesion. EMBO Rep. 2024;25(11):4803–26. doi: 10.1038/s44319-024-00261-z 39333626 PMC11549100

[52] Scarpa E, Mayor R. Collective cell migration in development. J Cell Biol. 2016;212(2):143–55. doi: 10.1083/jcb.201508047 26783298 PMC4738384

[53] Rock JR, Hogan BLM. Epithelial progenitor cells in lung development, maintenance, repair, and disease. Annu Rev Cell Dev Biol. 2011;27:493–512. doi: 10.1146/annurev-cellbio-100109-104040 21639799

[54] Huycke TR, Häkkinen TJ, Miyazaki H, Srivastava V, Barruet E, McGinnis CS, et al. Patterning and folding of intestinal villi by active mesenchymal dewetting. Cell. 2024;187(12):3072–89.e20. doi: 10.1016/j.cell.2024.04.039 38781967 PMC11166531

[55] Mongera A, Rowghanian P, Gustafson HJ, Shelton E, Kealhofer DA, Carn EK, et al. A fluid-to-solid jamming transition underlies vertebrate body axis elongation. Nature. 2018;561(7723):401–5. doi: 10.1038/s41586-018-0479-2 30185907 PMC6148385

[56] Chazaud C, Yamanaka Y, Pawson T, Rossant J. Early lineage segregation between epiblast and primitive endoderm in mouse blastocysts through the Grb2-MAPK pathway. Dev Cell. 2006;10(5):615–24. doi: 10.1016/j.devcel.2006.02.020 16678776

[57] McMahon AP. Development of the Mammalian Kidney. Curr Top Dev Biol. 2016;117:31–64. doi: 10.1016/bs.ctdb.2015.10.010 26969971 PMC5007134

[58] Sato T, Clevers H. Growing self-organizing mini-guts from a single intestinal stem cell: mechanism and applications. Science. 2013;340(6137):1190–4. doi: 10.1126/science.1234852 23744940

[59] Yang J, Chen J. Developmental programs of lung epithelial progenitors: a balanced progenitor model. Wiley Interdiscip Rev Dev Biol. 2014;3(5):331–47. doi: 10.1002/wdev.141 25124755 PMC4135449

[60] Scheele CLGJ, Hannezo E, Muraro MJ, Zomer A, Langedijk NSM, van Oudenaarden A, et al. Identity and dynamics of mammary stem cells during branching morphogenesis. Nature. 2017;542(7641):313–7. doi: 10.1038/nature21046 28135720 PMC6097610

[61] Varner VD, Nelson CM. Cellular and physical mechanisms of branching morphogenesis. Development. 2014;141(14):2750–9. doi: 10.1242/dev.104794 25005470 PMC4197615

[62] Plageman TF Jr, Chauhan BK, Yang C, Jaudon F, Shang X, Zheng Y, et al. A Trio-RhoA-Shroom3 pathway is required for apical constriction and epithelial invagination. Development. 2011;138(23):5177–88. doi: 10.1242/dev.067868 22031541 PMC3210497

[63] Pearl EJ, Li J, Green JBA. Cellular systems for epithelial invagination. Philos Trans R Soc Lond B Biol Sci. 2017;372(1720):20150526. doi: 10.1098/rstb.2015.0526 28348256 PMC5379028

[64] Huch M, Knoblich JA, Lutolf MP, Martinez-Arias A. The hope and the hype of organoid research. Development. 2017;144(6):938–41. doi: 10.1242/dev.150201 28292837

[65] Toda S, Blauch LR, Tang SKY, Morsut L, Lim WA. Programming self-organizing multicellular structures with synthetic cell-cell signaling. Science. 2018;361(6398):156–62. doi: 10.1126/science.aat0271 29853554 PMC6492944

[66] Gjorevski N, Sachs N, Manfrin A, Giger S, Bragina ME, Ordóñez-Morán P, et al. Designer matrices for intestinal stem cell and organoid culture. Nature. 2016;539(7630):560–4. doi: 10.1038/nature20168 27851739

[67] Beccari L, Moris N, Girgin M, Turner DA, Baillie-Johnson P, Cossy A-C, et al. Multi-axial self-organization properties of mouse embryonic stem cells into gastruloids. Nature. 2018;562(7726):272–6. doi: 10.1038/s41586-018-0578-0 30283134

[68] Merks RMH, Glazier JA. A cell-centered approach to developmental biology. Phys A Stat Mech Appl. 2005;352(1):113–30. doi: 10.1016/j.physa.2004.12.028

[69] Daub JT, Merks RMH. Cell-based computational modeling of vascular morphogenesis using Tissue Simulation Toolkit. Methods Mol Biol. 2015;1214:67–127. doi: 10.1007/978-1-4939-1462-3_6 25468600

[70] Reinitz J, Sharp DH. Mechanism of eve stripe formation. Mech Dev. 1995;49(1–2):133–58. doi: 10.1016/0925-4773(94)00310-j 7748785

[71] Kerridge S, Munjal A, Philippe J-M, Jha A, de las Bayonas AG, Saurin AJ, et al. Modular activation of Rho1 by GPCR signalling imparts polarized myosin II activation during morphogenesis. Nat Cell Biol. 2016;18(3):261–70. doi: 10.1038/ncb3302 26780298

[72] Lecuit T, Lenne P-F. Cell surface mechanics and the control of cell shape, tissue patterns and morphogenesis. Nat Rev Mol Cell Biol. 2007;8(8):633–44. doi: 10.1038/nrm2222 17643125

[73] Page DL, Koschan AF, Sukumar SR, Roui-Abidi B, Abidi MA. Shape analysis algorithm based on information theory. In: Proceedings 2003 International Conference on Image Processing (Cat. No.03CH37429). vol. 1. IEEE; 2003. p. I-229.

[74] Wuensche A. Basins of attraction in network dynamics. Modularity in Development and Evolution. 2004. p. 1–17.

