# Supplementary material for: Cell differentiation can underpin the reproducibility of morphogenesis
Source: PLoS Comput Biol. 2026 Jun 4;22(6):e1014361. doi: 10.1371/journal.pcbi.1014361 (PMC13252852; doi:10.1371/journal.pcbi.1014361)
Supplement: S1 Appendix — (PDF) [file pcbi.1014361.s001.pdf]

Appendix for: *Cell differentiation can underpin the reproducibility of morphogenesis*

Dominic K. Devlin [1,2*], Austen R.D. Ganley[1], Nobuto Takeuchi [1,3,4]

1School of Biological Sciences, The University of Auckland, Auckland, New Zealand
2 Graduate School of Arts and Sciences, The University of Tokyo, Tokyo, Japan
3 Universal Biology Institute, University of Tokyo, Tokyo 153-8902, Japan
4 Department of Biology, Faculty of Sciences, Kyushu University, Fukuoka, Japan

* dominicdevlin@g.ecc.u-tokyo.ac.jp

Morphologies with divisions of labour have higher reproducibility when accounting for differences in shape complexity. This section aims to determine whether the difference in reproducibility scores between evolved morphologies with and without morphogenetic divisions of labour can be attributed to variations in their shape complexity. Morphogenetic divisions of labour are defined by the presence of multiple SCCs, with moving and stationary cell states separated into distinct SCCs (see main text). To demonstrate the relationship between shape complexity and reproducibility in our model, imagine the simplest morphological shape: one that remains circular over development. The reproducibility of this morphology will be trivially high because its circular morphology will not change in each developmental replicate. In contrast, morphogenesis requires extensive cell motion. Extensive cell motion is prone to noise in cell motion and geometry, which means that morphogenesis tends to become more susceptible to noise with increasing complexity of these cell movements. This relationship between complexity and reproducibility has also been demonstrated in a previous study [1].

To answer whether differences in reproducibility scores between evolved morphologies with and without divisions of labour can be attributed to variations in their shape complexity, we first compared the average shape complexity between the two groups using the technique outlined in Materials and methods, averaged over 60 developmental replicates. We found that those with divisions of labour ($\mu = 94.1$, $\sigma = 13.3$) exhibit lower shape complexity on average than those without ($\mu = 116.5$, $\sigma = 18.0$), and that this difference was statistically significant ($p = 10^{-4}$, two-tailed t-test). Although our quantification of shape complexity is arbitrary, this result suggests that the difference in reproducibility scores may in part be explained by differences in shape complexity.

We conducted two further analyses to more rigorously determine whether this difference in shape complexity was responsible for the difference in reproducibility scores. For the first analysis, we examined whether "group similarity" differed significantly between morphologies with and without morphogenetic divisions of labour. Group similarity measures how similar the morphologies are within a group (with or without progenitor-cell differentiation). To quantify group similarity, we computed the shape similarity of one developmental replicate of each morphology within a group at 12,000 developmental time steps (DTS) to all others within the same group using the same overlap method we used to determine reproducibility scores (Materials and methods). Suppose the morphologies within a group have low complexity. In this case, they will deviate little from the initial circular shape, resulting in higher morphological similarity and, thus, higher group similarity scores. Conversely, more complex morphologies will result in morphological dissimilarity and, thus, lower group similarity scores. The advantage of using group similarity to determine the link between reproducibility and complexity is that it can be compared to reproducibility scores, as it uses the same technique, while avoiding our arbitrary quantification of shape complexity. The results show that morphologies with divisions of labour had a marginally higher group similarity (mean=45.5%) than morphologies without (mean=43.9%, $p = 0.036$ two-tailed t-test; S10 Fig panel B). Despite this marginally higher similarity, there was a significant overlap in the interquartile ranges between the two groups. Moreover, the difference in means of group similarity scores is much smaller than the difference in reproducibility scores, which were, on average, 72.1% for morphologies with divisions of labour versus 52.0% for morphologies without ($p < 10^{-12}$, two-tailed t-test). This result indicates that the difference in shape complexity between the groups does not fully account for their reproducibility differences.

For the second analysis, we conducted a regression to examine whether the relationship between shape complexity and reproducibility differs between those with and without morphogenetic divisions of labour. The regression model we used is:

$$R_i = \beta_0 + \beta_1 \cdot M_i + \beta_2 \cdot (M_i \times \text{DOL}_i) + \epsilon_i \tag{1}$$

where M_i and R_i are the shape complexity and reproducibility of morphology i, respectively. The term "DOL_i" evaluates to one if morphology i has a division of labour; otherwise, zero. We found that reproducibility declines more rapidly as complexity increases for those without divisions of labour (95% confidence interval on β_1 is $[-3.0 \times 10^{-3}, -1.3 \times 10^{-3}]$, $n = 65$), compared to those with them (95% confidence interval on $\beta_1 + \beta_2$ is $[-1.2 \times 10^{-3}, 1.9 \times 10^{-5}]$, $n = 24$). Moreover, when morphologies are matched for shape complexity, the graph shows no obvious overlap between the two groups (S10 Fig panel C). This result indicates that the elevated reproducibility observed in morphologies with divisions of labour is not because of their lower shape complexity than those without such systems.

The above result that reproducibility declines less with complexity in morphologies that have divisions of labour compared to those without indicates that division of labour allows morphogenesis to bypass a reproducibility-complexity trade-off. Ascertaining the existence of this trade-off from our regression analysis is difficult because the sample sizes we used are small (only 90 evolved morphologies). Therefore, we increased our sample size by taking samples of many morphologies from each simulation. We measured the reproducibility and average shape complexity of the morphologies with the highest fitness in each population at 100-generation intervals throughout each simulation. We analysed 36 simulations: the 18 that evolved morphogenetic divisions of labour and 18 that did not (we chose the latter based on having similar endpoint shape complexity to the former). We performed a linear regression of reproducibility against shape complexity for each simulation separately. The results show that reproducibility declined with complexity in every simulation (S10 panel D), indicating that morphologies become less reproducible as they became more complex. However, the slopes appear steeper in simulations where poorly reproducible morphologies evolved. To quantify this, we bootstrapped the coefficients of the linear regression slopes to obtain a confidence interval of this coefficient for the 18 simulations where morphogenetic division of labour evolved and the 18 where they didn't. We found that this coefficient was much smaller in simulations where morphogenetic division of labour evolved (95% CI of slope is -0.0017 to -0.0011, S10 Fig panel D, blue lines) compared to simulations where it did not (95% CI of slope is -0.0035 to -0.0029, S10 Fig Panel D, orange lines), indicating that reproducibility declines more rapidly as complexity increases without morphogenetic division of labour. Moreover, the ability of shape complexity to predict a morphologies' reproducibility was much weaker in those that evolved morphogenetic division of labour (average $R^2 = 0.59$ across the 18 simulations), compared to those that did not (average $R^2 = 0.91$ across the 18 simulations). These findings support the hypothesis that morphogenesis via a division of labour bypasses a trade-off between shape complexity and reproducibility. Moreover, the fact that differences in reproducibility persist through evolution suggests that simulations where divisions of labour evolved are on different evolutionary trajectories than those where they did not (these trajectories are likely determined by the initial conditions or early in the evolutionary simulations).

Summary. Together, our results show that differences in complexity are insufficient to account for the high reproducibility observed in morphologies associated with progenitor-cell differentiation. The first result supporting this claim is that the difference in group similarity between morphologies with progenitor-cell differentiation and those without is much smaller than the difference in reproducibility between groups (S10 Fig panels A and B). Since group similarity inversely correlates with shape complexity, this result indicates that differences in complexity do not account for differences in reproducibility between groups. Second, when we performed a regression analysis of reproducibility scores on shape complexity, the regression slope was much steeper in morphologies without progenitor-cell differentiation than in those with it (S10 Fig panels C and D). This result indicates that morphologies with progenitor-cell differentiation almost always exhibit higher reproducibility than those without when matched for shape complexity (S10 Fig panel A). Third, both high morphogenetic reproducibility and high shape complexity are almost exclusively observed in morphologies with progenitor-cell differentiation, even under different selection pressures (S2 Fig).

Morphogenetic divisions of labour can evolve from most initial gene regulatory networks. Our results show that morphogenetic divisions of labour with progenitor-cell differentiation evolved only in a minority of simulations in the main text (24 out of the 90 morphologies with complex shapes). We asked whether this frequency is because progenitor-cell differentiation evolve from only a restricted portion of genotype space, and thus determined by the initial conditions, or whether the frequency depends on the selection pressure. To address this, we tested an alternative selection criterion that favoured not only the shape complexity but also the directional motion of morphologies. This additional selection for directional motion is expected to favour progenitor-cell differentiation because directional motion is a common property of these morphologies (Fig 5F in the main text), although it does not directly select for progenitor-cell differentiation or reproducibility. We quantified directional motion by determining how much a morphologies' centre of mass (measured in pixels) shifts over the 12,000 DTS. The selection pressure we used was an additive combination of this directional motion and the original quantification of shape complexity (Materials and methods). We ran 35 simulations lasting at least 2.5×10^3 evolutionary generations, of which 31 evolved morphologies surpassed our arbitrary threshold of shape complexity (Materials and methods). We found the great majority of these morphologies (29 out of 31) had evolved progenitor-cell differentiation (S2 Fig). Of

these 29 morphologies, all but one displayed highly reproducible morphogenesis (S2 Fig panel D). In contrast, the two morphologies that did not evolve progenitor-cell differentiation displayed poorly reproducible morphogenesis (S2 Fig panels D and H). In simulations where we only selected for directional motion and not shape complexity, progenitor-cell differentiation evolved in seven out of 25 simulations (S12 Fig panel A). These results show that progenitor-cell differentiation can evolve from most initial gene regulatory networks.

References

1. Hagolani PF, Zimm R, Marin-Riera M, Salazar-Ciudad I. Cell signaling stabilizes morphogenesis against noise. Development. 2019;146(20):dev179309.
